# Three Sulfated Triterpene Glycosides from the Sea Cucumber *Psolus phantapus*—Biological Activity Against Human Cancer Cell Lines

**DOI:** 10.3390/md24060202

**Published:** 2026-06-08

**Authors:** Alexandra S. Silchenko, Ekaterina A. Chingizova, Ekaterina S. Menchinskaya, Kseniya M. Tabakmakher, Anatoly I. Kalinovsky, Sergey A. Avilov, Roman S. Popov, Vadim G. Stepanov, Vladimir I. Kalinin

**Affiliations:** 1 G.B. Elyakov Pacific Institute of Bioorganic Chemistry, Far Eastern Branch of the Russian Academy of Sciences, Pr. 100-letya Vladivostoka 159, 690022 Vladivostok, Russia; chingizova_ea@piboc.dvo.ru (E.A.C.); ekaterinamenchinskaya@gmail.com (E.S.M.); tabakmakher_km@piboc.dvo.ru (K.M.T.); kaaniv@piboc.dvo.ru (A.I.K.); avilov_sa@piboc.dvo.ru (S.A.A.); popov_rs@piboc.dvo.ru (R.S.P.); kalininv@piboc.dvo.ru (V.I.K.); 2 Kamchatka Branch of Pacific Institute of Geography, Far Eastern Branch of the Russian Academy of Sciences, Partizanskaya St. 6, 683000 Petropavlovsk-Kamchatsky, Russia; stepanovvadim24@gmail.com

**Keywords:** *Psolus phantapus*, Dendrochirotida, triterpene glycosides, sea cucumber, hemolytic and cytotoxic activity, human breast cancer

## Abstract

The glycosidic composition of *Psolus phantapus* was studied for the first time. Two new glycosides, phantapusosides A (**1**) and B (**2**), and the known psolusoside P (**3**) were isolated and their structures were established by analysis of ^1^H, ^13^C NMR, 1D TOCSY, and 2D NMR (^1^H,^1^H COSY, HMBC, HSQC, ROESY), and HR-ESI mass spectra. These compounds are structurally close to those isolated from other representatives of the genus *Psolus*: *P. fabricii*, *P. peronii* and *P. chitonoides*. These data confirm the chemotaxonomic significance of triterpene glycosides of sea cucumbers, demonstrating that closely related species biosynthesize structurally similar metabolites. The cytotoxic activity of compounds **1** and **2** was studied against four human breast cancer cell lines (MCF-7, T-47D, MDA-MB-231, MDA-MB-468), as well as the non-tumorigenic mammary epithelial cell line MCF-10A and the pancreatic epithelioid carcinoma cell line PANC-1. The glycosides were selectively active against the TNBC cell lines MDA-MB-231 and MDA-MB-468. Notably, both glycosides inhibited the clonogenic potential of TNBC cell lines more significantly than their metabolic activity (MTT assay) and demonstrated a more pronounced colony-inhibiting effect toward the basal-like cell line MDA-MB-468, making this cell line a promising model for future investigation of the antitumor effects of glycosides.

## 1. Introduction

Marine invertebrates belonging to the class Holothuroidea are known as sea cucumbers or holothurians. They have attracted significant scientific interest over several decades due to their rich content of bioactive compounds, particularly triterpene glycosides. These secondary metabolites, also known as saponins, exhibit a wide range of pharmacological properties, including cytotoxic, antifungal, hemolytic, immunomodulatory [1,2,3,4,5,6], and anticancer activities [7,8,9,10,11]. Triterpene glycosides of sea cucumbers are characterized by unique aglycone structures, derived from lanostane, and oligosaccharide chains consisting of two to six sugar moieties that contribute to their biological activity and taxonomic specificity [12,13,14]. A distinctive feature of many holothurious glycosides is the presence of sulfate groups (from one to four) attached to different monosaccharide residues, which also affects their bioactivity and polarity.

The aglycones consist of a tetra- (non-holostane aglycones without a lactone) or pentacyclic (holostane-type aglycones with 18(20)-lactone, or non-holostane aglycones with 18(16)-lactone) backbone, with structural modifications such as the presence of a 7(8)-, 9(11), or rarely 8(9)-double bond, and various oxygen-containing functional groups (e.g., hydroxyl, keto-, epoxy- and acetate groups). The carbohydrate chains, always attached at the C-3 position of aglycones, vary in length, architecture and composition, commonly including xylose, quinovose, glucose, and 3-O-methylated glucose, xylose or even quinovose. This structural diversity leads to a wide array of compounds with distinct biological properties, providing an opportunity to study the structure–activity relationships (SARs), including the application of the modern quantitative structure–activity relationship (QSAR) technique [15,16].

The study of holothurian triterpene glycosides is of great importance for both chemotaxonomy [12,13,14] and drug discovery. The structural diversity of these compounds makes it possible to distinguish species and genera, while their potent bioactivity makes them promising candidates for the development of novel therapeutics. Nevertheless, despite extensive research on glycosides, several related issues remain unresolved. Among these are the functional characteristics of the genes and enzymes participating in glycoside biosynthesis, as well as ecological aspects, including variability in glycoside composition in relation to the geographical distribution of the species and seasonal changes.

A notable example of structural diversity is the series of triterpene glycosides from sea cucumbers of the genus *Psolus*. This genus comprises 58 species of sea cucumbers, five of which have been studied chemically: *P. fabricii* [17,18,19], *P. eximius* [20], *P. patagonicus* [21], *P. chitonoides* [22,23,24] and *P. peronii* [25]. These species produce glycosides with highly oxidized aglycones, containing all three possible positions of the intranuclear double bond (7(8)-, 8(9)- and 9(11)-); hydroxy-, epoxy-, keto-, or *O*-acetic functionalities in the polycyclic systems; and hydroxy-, keto- and peroxy-groups in the side chains. The aglycones of glycosides of *Psolus* species are related to the holostane type (with 18(20)-lactone) and to the non-holostane type (with 18(16)-lactone or the unique 18(20)-epoxy-group). Carbohydrate chains also contribute to chemical diversity due to their uncommon architecture (tetrasaccharide chains containing two units each in the upper and lower semi-chains and rare trisaccharide sugar moieties in the glycosides of *P. fabricii* [17,18], or three units in the upper and one unit in the lower semi-chain in the glycosides of *P. chitonoides* [22,23]), their sugar composition characterized by the presence of rare 3-*O*-methylxylose as a terminal unit [22,23], or glucose or xylose as the second sugar moiety in the chains [17,18,19,20,25]. However, the most significant distinguishing features of these compounds are the positions and number of sulfate groups. In *P. fabricii,* the glycosides bearing sulfate groups at C-2 and C-4 of the glucose unit have been found, while the common position of sulfation is the hydroxymethylene group (C-6) of hexoses. Moreover, the simultaneous attachment of two sulfate groups to a single monosaccharide residue is also a unique structural characteristic [18,25]. In *P. chitonoides*, and subsequently in *P. fabricii,* tetrasulfated glycosides were discovered, for the first time, among sea cucumbers [18,24].

In continuation of our investigations of glycosides from the sea cucumbers belonging to the genus *Psolus,* we isolated three triterpene glycosides from *Psolus phantapus*, including new phantapusosides A (**1**) and B (**2**) and known psolusoside P (**3**), found earlier in *Psolus fabricii* [18]. Their structures were elucidated by extensive analyses of ^1^H, ^13^C NMR, 1D TOCSY, and 2D NMR (^1^H,^1^H COSY, HMBC, HSQC, ROESY), and HR-ESI mass spectra. All original spectra are provided in Figures S1–S18 in the Supplementary Data. The hemolytic activity against human erythrocytes and the cytotoxicity against human breast cancer cell lines MCF-7, T-47D, triple-negative MDA-MB-231 and MDA-MB-468, and the non-tumorigenic mammary epithelial cell line MCF-10A, as well as against pancreatic carcinoma PANC-1 cells, were tested for new compounds **1** and **2**.

## 2. Results and Discussion

### 2.1. Structure Elucidation of Glycosides

**Figure 1:**
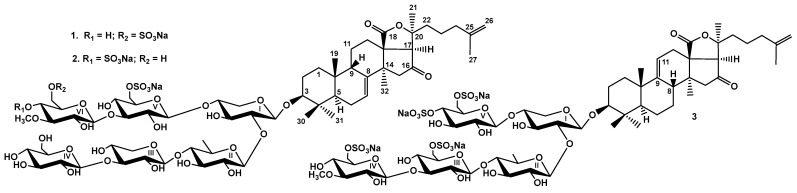
Chemical structures of the glycosides from *Psolus phantapus*: **1**—phantapusoside A; **2**—phantapusoside B; **3**—psolusoside P.

Specimens of *P. phantapus* (five pieces) were collected from Avacha Bay by scuba diving. Glycosides were isolated from the concentrated ethanolic extract which was previously subjected to hydrophobic chromatography on Polychrom-1-eluting glycosides with 50% EtOH followed by silica gel column chromatography with solvent system CHCl_3_/EtOH/H_2_O (4/5/1) as the mobile phase, giving a total fraction weighing 51.5 mg. Such a small total glycosidic weight did not allow us to elucidate the structures of some minor compounds, because their weights were insufficient for NMR spectra registration. So, only three glycosides **1**–**3** (Figure 1) were isolated by subsequent HPLC on reversed-phase columns in acceptable amounts for structure elucidation.

The monosaccharides composing the glycosides of *P. phantapus* were assigned to the D-series based on biogenetic considerations, since the D-configuration has been experimentally established for all monosaccharide residues of the glycosides of *Psolus fabricii*, including psolusoside P (**3**) [26,27]. Furthermore, all known sea cucumber triterpene glycosides are characterized by the D-configuration of their monosaccharide constituents.

The molecular formula of phantapusoside A (**1**) was determined to be C_65_H_100_O_37_S_2_Na_2_ from the [M_2Na_ − Na]^−^ ion peak at *m*/*z* 1559.5249 (calc. for C_65_H_100_O_37_S_2_Na as 1559.5288, Δ 2.5 ppm) and the [M_2Na_ − 2Na]^2−^ ion peak at m/z 768.2699 (calc. for C_65_H_100_O_37_S_2_ as 768.2698, Δ −0.1 ppm) in (*−*)HR-ESI-MS (Figure S8). As deduced from extensive analysis of the NMR spectra, the aglycone of **1** was a holostanetype, i.e., contained 18(20)-lactone (from the signals of C-18 at *δ*_C_ 179.3 and C-20 at *δ*_C_ 83.8), as well as a 7(8)-double bond (from the signals at *δ*_C_ 121.7 (C-7), 143.9 (C-8) and the corresponding proton at *δ*_H_ 5.66 m (H-7) in the ^13^C and ^1^H NMR spectra), 25(26)-double bond (from the signals at *δ*_C_ 145.5 (C-25), *δ*_C_ 110.4 (C-26) and 4.69 (H-26′, s) and 4.68 (H-26′′, s) in the ^13^C and ^1^H NMR spectra, correspondingly), and 16-keto group (from the signal at *δ*_C_ 214.2 (C-16) (Table 1, Figures S1–S6)). As the spatial structure of holostane-type aglycones is well-established, the orientation of methyl (CH_3_-19, CH_3_-21, CH_3_-30, CH_3_-31, CH_3_-32) as well as the characteristic configuration of the C-9 chiral center of the sea cucumber glycosides were confirmed by NOE-correlations: H-19/H-9, H-30; H-21/H-17; H-31/H-3, H-5; H-32/H-7 and H-9/H-19 observed in the spectrum of **1** (Table 1). Hence, the structure of the aglycone part of **1** was identified as holosta-7,25-dien-3β-ol-16-one, which was found first in cucumarioside A_2_-2, the main component of the glycosidic fraction of *Cucumaria japonica* [28], and is common for sea cucumbers.

Analysis of the ^1^H, ^13^C NMR, and HSQC spectra of the carbohydrate moiety of phantapusoside A (**1**) (Table 2; Figures S1–S7) revealed the presence of six doublets of the anomeric protons at *δ*_H_ 4.66–5.22 (*J* = 7.5–8.1 Hz) and the signals of the corresponding anomeric carbons at *δ*_C_ 102.2–104.8, indicating the presence of a hexasaccharide chain with *β*-glycosidic bonds [29]. The first monosaccharide residue is always attached to C-3 of the aglycone. So, the anomeric proton of the first sugar unit at *δ*_H_ 4.66 (H-1 Xyl1, d, *J* = 7.5 Hz) was deduced from the NOE correlation of H-3 of the aglycone at *δ*_H_ 3.19 (H-3, dd, *J* = 11.8; 3.4 Hz) with H-1 Xyl1. The subsequent analysis of the ^1^H, ^1^H COSY, 1D TOCSY, HSQC, and ROESY spectra allowed for the establishment of the structure of this monosaccharide as xylose (Xyl1). The signals of C-2 and C-4 Xyl1 were deshielded to 82.0 and 77.9, respectively, indicating the glycosylation of these positions. Indeed, further analysis of the ROESY spectrum revealed the correlations H-2 Xyl1/H-1 Qui2 and H-4 Xyl1/H-1 Glc5, which confirmed the positions of glycosidic bonds. Additionally, coupling patterns of protons and NOE correlations H-1/H-3, H-1/H-5, and H-3/H-5 of the xylose, glucose and 3-O-methylglucose, as well as H-2/H-4 of quinovose (Table 1), unambiguously indicate the nature of monosaccharides. It was concluded that the oligosaccharide moiety of **1** consisted of two xyloses (as the first and third units in the chain), quinovose (as the second unit), glucose (as the fourth and fifth residues) and 3-*O*-methylglucose (as the terminal (sixth) unit). Analysis of NOE and HMBC correlations showed the positions of glycosidic bonds. Correlations were observed between the H-1 Xyl1 and H-3 (C-3) of the aglycone, H-1 Qui2 and H-2 (C-2) Xyl1, H-1 Xyl3 and H-4 (C-4) Qui2, H-1 Glc4 and H-3 (C-3) Xyl3, H-1 Glc5 and H-4 (C-4) Xyl1, and H-1 MeGlc6 and H-3 (C-3) Glc5 (Table 2). Notably, that terminal sugar residue in the lower semi-chain was non-methylated glucose, which is a rare structural feature. The presence of two sulfate groups was deduced from HR-ESI-MS data and also confirmed by the ^13^C NMR spectrum of **1**. Their positions were established based on *α*- and *β*-shifting effects, which were observed for the signals of C-6 Glc5 (*δ*_C_ 68.2) and C-5 Glc5 (*δ*_C_ 76.0), and for C-6 MeGlc6 (*δ*_C_ 67.8) and C-5 MeGlc5 (*δ*_C_ 76.5), correspondingly [29]. By contrast, the signals of non-sulfated carbons of hydroxymethylene groups of hexoses (C-6) are usually observed at *δ*_C_ ~ 61.0–61.3, and the adjacent C-5 signals are usually observed at *δ*_C_ ~ 77.0–77.5 [18,19,22,24]. Thus, the sulfate groups were attached to C-6 of Glc5 and C-6 of MeGlc6 in phantapusoside A (**1**).

**Figure 2:**
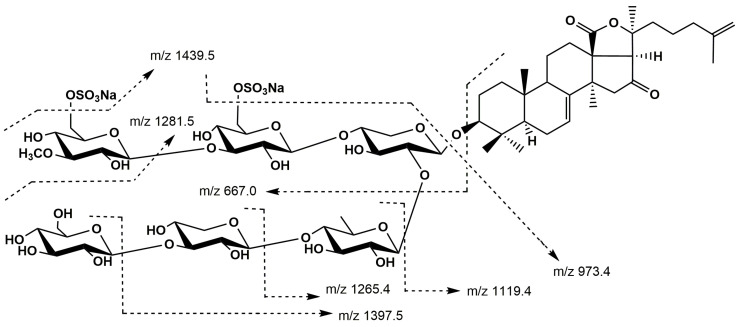
The scheme of phantapusoside A (**1**) fragmentation in the (−)ESI-MS/MS spectrum.

The (*−*)ESI-MS/MS of **1** (Figure S8) demonstrated the fragmentation of the [M_2Na_ − Na]^−^ ion at *m*/*z* 1559.5, leading to the appearance of fragment ions at *m*/*z* 1439.5 [M_2Na_ − Na − NaHSO_4_]^−^, 1397.5 [M_2Na_ − Na − Glc]^−^, 1281.5 [M_2Na_ − Na − MeGlcSO_3_Na]^−^, 1265.4 [M_2Na_ − Na − Glc − Xyl]^−^, 1119.4 [M_2Na_ − Na − Glc − Xyl − Qui]^−^, 999.4 [M_2Na_ − Na − Glc − Xyl − Qui − NaHSO_4_]^−^, 973.4 [M_2Na_ − Na − Agl − NaSO_4_]^−^, and 667.0 [M_2Na_ − Na − Agl − Glc − Xyl − Qui − H]^−^, corroborating the structure of both the sugar chain and aglycone of **1** (Figure 2).

These data indicate that phantapusoside A (**1**) is 3*β*-*O*-{*β*-D-glucopyranosyl-(1→3)-*β*-D-xylopyranosyl-(1→4)-*β*-D-quinovopyranosyl-(1→2)-[6-*O*-sodium sulfate-3-*O*-methyl-*β*-D-glucopyranosyl-(1→3)-6-*O*-sodium sulfate-*β*-D-glucopyranosyl-(1→4)]-*β*-D-xylopyranosyl}-16-keto-holosta-7(8),25-diene.

The molecular formula of phantapusoside B (**2**) was determined to be C_65_H_100_O_37_S_2_Na_2_ from the [M_2Na_ − Na]^−^ ion peak at *m*/*z* 1559.5281 (calc. for C_65_H_100_O_37_S_2_Na as 1559.5288, Δ 0.4 ppm), and from the [M_2Na_ − 2Na]^2−^ ion peak at *m/z* 768.2702 (calc. for C_65_H_100_O_37_S_2_ as 768.2698, Δ −0.5 ppm) in (*−*)HR-ESI-MS (Figure S16). The coincidence of molecular formulae of **1** and **2** indicated their isomerism. The aglycone of **2** was the same as in **1**, as deduced from their identical NMR spectra (Table S1, Figures S9–S14). Hence, the structural differences of these glycosides were attributed to the carbohydrate chains.

Analysis of the ^1^H, ^13^C NMR, and HSQC spectra of the carbohydrate moiety of phantapusoside B (**2**) (Table 3; Figures S9–S15) also demonstrated the presence of six doublets of the anomeric protons at *δ*_H_ 4.66–5.19 (*J* = 7.2–8.6 Hz) and the signals of the corresponding anomeric carbons at *δ*_C_ 102.2–104.8. Thus, analogously to **1**, the carbohydrate chain of **2** consisted of six monosaccharide residues linked through *β*-glycosidic bonds. Applying the same algorithm of analysis of NMR data, it was concluded that the oligosaccharide moiety of **2** had the same sugar composition and architecture, arising out of the positions of glycosidic linkages, which glycoside **1** had. Therefore, the difference could only lie in the position of sulfate groups. The comparison of chemical shifts of sulfated monosaccharide residues in **1** and **2** showed the varying values only for terminal 3-*O*-methylglucose. The signals of C-4 MeGlc6 and C-6 MeGlc6 in the spectrum of **2** were shifted downfield to *δ*_C_ 76.2 and upfield to *δ*_C_ 61.8, respectively, as compared with the corresponding values at *δ*_C_ 70.6 (C-4 MeGlc6) and *δ*_C_ 67.8 (C-6 MeGlc6) for **1**. These data indicated the bonding of the sulfate group to C-4 MeGlc6 in **2**, whose signal was deshielded due to an *α*-shifting effect [29]. Meanwhile, the signal of C-6 Meglc6 in the spectrum of **2** was shielded due to the absence of a sulfate group. The second sulfate group occupied the same position as in **1**—at C-6 Glc5 (*δ*_C_ 67.2). The glycosides with the sulfate group attached to C-4 of monosaccharide residues were first discovered relatively recently (in 2017) in the sea cucumber *Stichopus horrens* [30], and were then isolated from *Psolus fabricii* [18], *P. chitonoides* [22,23,24], *Colochirus quadrangularis* [31], and *Paracaudina chilensis* [32]. The carbohydrate chain of phantapusoside B (**2**) turned out to be the same as the chain of chitonoidoside I from *P. chitonoides* [24]. The comparison of the ^13^C NMR spectra of sugar parts of phantapusoside B (**2**) and chitonoidoside I showed the proximity of corresponding *δ*_C_ values that confirmed their identity.

The (*−*)ESI-MS/MS spectrum of **2** (Figure S16) demonstrated the fragmentation of the [M_2Na_ − Na]^−^ ion at *m*/*z* 1559.5, leading to the appearance of a number of the same fragment ions as in the MS/MS spectrum of **1** at *m*/*z*: 1439.5 [M_2Na_ − Na − NaHSO_4_]^−^, 1281.5 [M_2Na_ − Na − MeGlcSO_3_Na]^−^, 1119.4 [M_2Na_ − Na − Glc − Xyl − Qui]^−^, and 667.0 [M_2Na_ − Na − Agl − Glc − Xyl − Qui − H]^−^. Additional ion peaks were deciphered from the (*+*)HR-ESI-MS/MS, which were observed at *m/z* 1485 [M_2Na_ + Na − NaHSO_4_]^+^, 1327 [M_2Na_ + Na − MeGlcSO_3_]^+^, 1165 [M_2Na_ + Na − MeGlcSO_3_ − Glc]^+^, 1045 [M_2Na_ + Na − MeGlcSO_3_ − GlcSO_3_]^+^, and 697 [M_2Na_ + Na − Agl − Glc − Xyl − Qui − H]^+^, corroborating the structure of **2**.

These data indicate that phantapusoside B (**2**) is 3*β*-*O*-{*β*-D-glucopyranosyl-(1→3)-*β*-D-xylopyranosyl-(1→4)-*β*-D-quinovopyranosyl-(1→2)-[4-*O*-sodium sulfate-3-*O*-methyl-*β*-D-glucopyranosyl-(1→3)-6-*O*-sodium sulfate-*β*-D-glucopyranosyl-(1→4)]-*β*-D-xylopyranosyl}-16-keto-holosta-7(8),25-diene.

The molecular formula of glycoside **3** was determined to be C_60_H_90_O_39_S_4_Na_4_ from the [M_4Na_ − Na]^−^ ion peak at *m*/*z* 1631.3648 (calc. for C_60_H_90_O_39_S_4_Na_3_ as 1631.3641, Δ −0.4 ppm), the [M_4Na_ − 2Na]^2−^ ion peak at *m/z* 804.1873 (calc. for C_60_H_90_O_39_S_4_Na_2_ as 804,1873, Δ 0.1 ppm), the [M_4Na_ − 3Na]^3−^ ion peak at *m/z* 528.4616 (calc. for C_60_H_90_O_39_S_4_Na as 528.4619, Δ 0.6 ppm), and the [M_4Na_ − 4Na]^4−^ ion peak at *m/z* 390.5987 (calc. for C_60_H_90_O_39_S_4_ as 390.5991, Δ 1.0 ppm) in (*−*)HR-ESI-MS (Figure S18). The ^13^C NMR spectrum of **3** (Figure S17) was coincident with that of psolusoside P (**3**), isolated earlier from *Psolus fabricii* [18] and *P. peronii* [25]. It was the first tetrasulfated glycoside that was found with two sulfate groups connected to one monosaccharide residue.

Thus, the glycosides isolated from the sea cucumber *P. phantapus* are structurally close to those isolated from the other representatives of genus *Psolus*: *P. fabricii*, *P. peronii* and *P. chitonoides*. They have the same aglycones as the glycosides of both of the latter species; phantapusoside B (**2**) possesses a carbohydrate chain identical to that of chitonoidoside I, and finally *P. fabricii*, *P. peronii* and *P. phantapus* all contain psolusoside P (**3**). These data further demonstrate the chemotaxonomic significance of triterpene glycosides of the sea cucumbers, showing that closely related species biosynthesize structurally similar metabolites.

### 2.2. Biological Activity of Phantapusosides A (***1***) and B (***2***)

The cytotoxic activity of the compounds **1** and **2** was studied against four types of human breast cancer cells (MCF-7, T-47D, and two triple-negative (TNBC) lines MDA-MB-231 (mesenchymal-like) and MDA-MB-468 (basal-like)), as well as the non-tumorigenic mammary epithelial cell line MCF-10A and pancreatic epithelioid carcinoma PANC-1 cell line. Cucumarioside A_0_-1 [11] was used as the positive control. Cytotoxic activity against all the selected cell lines was assessed using the MTT method (Table 4).

Phantapusosides A (**1**) and B (**2**) exhibited strong hemolytic activity against human erythrocytes, indicating membranolytic action due to binding to membrane cholesterol and lipids [15]. In contrast, their cytotoxic activity against cancer cells was comparatively lower, although some selectivity of the glycosides towards different cancer cell lines was observed. The TNBC cell lines MDA-MB-231 and MDA-MB-468, and to a lesser extent the pancreatic cancer cell line PANC-1, were more sensitive to the cytotoxic action of the glycosides than the other tested cell lines.

As previously reported, MDA-MB-231 cells were more sensitive to the cytotoxic action of the glycosides, while the MCF-7 and T-47D cell lines were more resistant [33,34,35]; the same was observed for compounds **1** and **2**. It has been established that, beyond their interaction with membrane lipids, the glycosides appear to target the adenosine receptor subtype A_2B_ (A_2B_AR), which is highly expressed on MDA-MB-231 cells. The sea cucumber glycosides act as functionally selective antagonists of A_2B_AR, exhibiting signaling bias towards the inhibition of the MAPK pathway—a process underlying the antitumor action of the glycosides [36,37]. Thus, the higher activity of **1** and **2** against MDA-MB-231, MDA-MB-468 and PANC-1, in comparison with MCF-7 and T-47D, can be explained by the increased expression of A_2B_AR on these cells. However, the cytotoxicity of phantapusosides A (**1**) and B (**2**) against MDA-MB-231 cells was considerably lower than that of the lead glycosides (okhotoside A_1_-1 and cucumarioside A_0_-1 (used as positive control)) selected earlier from a large series of tested glycosides [11,34,35]. This can be explained by the presence of bulky hexasaccharide chains in **1** and **2**, which limit their incorporation into the membrane. This is in good agreement with the previous findings, where the glycosides with linear tetrasaccharide chains and holostane-type aglycones are generally the most active [15,16].

Notably, compound **2** was more active than compound **1**, and its IC_50_ was close to that of cucumarioside A_0_-1 (positive control). Since glycoside **2** differed from **1** by the attachment of the sulfate group at C-4 MeGlc6 instead of C-6 MeGlc6, the difference in their activity was directly related to this structural feature, which has been shown to strongly influence the cytotoxic properties of the glycosides [15]. Additionally, the nearly identical hemolytic ED_50_ values of **1** and **2** (Table 4) contrast with their divergent anticancer cytotoxicity, implying that the differential positioning of the sulfate group primarily modulates the receptor-mediated antitumor mechanisms rather than general membranolytic properties.

**Figure 3:**
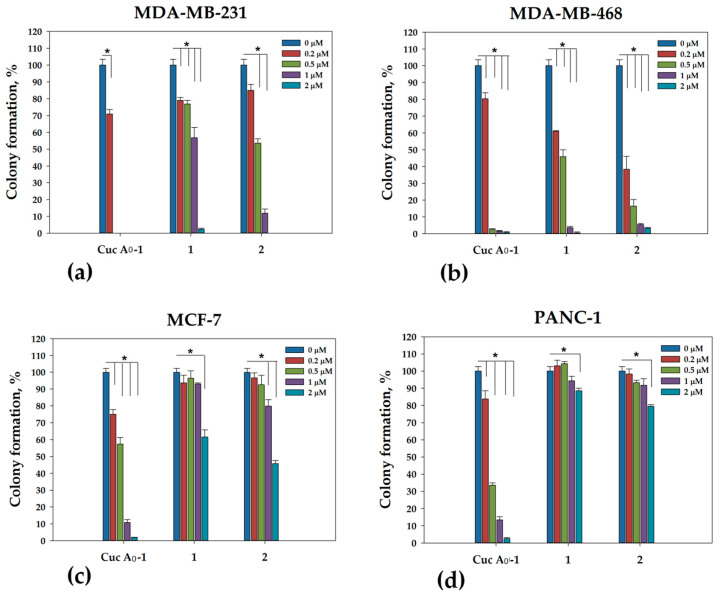
Colony formation by MDA-MB-231 (**a**), MDA-MB-468 (**b**), MCF-7 (**c**) and PANC-1 (**d**) cells under the action of glycosides **1** and **2** and Cuc A_0_-1 in different concentrations. Data are presented as means ± SEM. * *p* value < 0.05 considered significant.

The reproductive integrity of a single cell—its capacity to divide and produce a visible colony—forms the basis of the colony formation assay, a standard tool for in vitro survival studies. This phenomenon directly mirrors the in vivo situation, where the unrestrained proliferative potential of malignant cells is responsible for the development of metastases. To study the effect of the tested glycosides on the formation and growth of tumor cell colonies, a range of non-toxic concentrations was used against the MDA-MB-231, MDA-MB-468, MCF-7 and PANC-1 cell lines (Figure 3). A dose-dependent effect of colony growth inhibition was observed for both compounds, but it was less pronounced in relation to MCF-7 and PANC-1 lines. Phantapusoside B (**2**) more strongly inhibited colony formation and growth than phantapusoside A (**1**) (Table 5).

At a concentration of 0.5 μM, compound **2** demonstrated inhibition of MDA-MB-231 cells by 47% and MDA-MB-468 cells by 84%. The increased concentration of **2** (1 μM) led to a stronger inhibition of colony formation of MDA-MB-231 cells by 88% and of MDA-MB-468 cells by 95%. The dosage of 2 μM of phantapusoside B (**2**) almost completely blocked colony formation of both the MDA-MB-231 and MDA-MB-468 lines, and caused 54% of growth inhibition of MCF-7 cell colonies (Figure 3).

Compound **1** at a concentration of 0.5 μM inhibited colony formation of MDA-MB-231 cells by 23% and MDA-MB-468 cells by 54%, at a concentration of 1 μM—by 43% and 94%, respectively. An amount of 2 μM of phantapusoside A (**1**) acted similarly to glycoside **2**, almost completely blocking colony formation of MDA-MB-231 and MDA-MB-468 lines, and causing 39% of growth inhibition of MCF-7 cell colonies (Figure 3).

Notably, both glycosides (**1**, **2**) inhibited the clonogenic potential of TNBC cell lines more significantly than their metabolic activity (MTT assay). They demonstrated a more pronounced colony-inhibiting effect toward the basal-like cell line MDA-MB-468. Colony formation and growth of PANC-1 cells were not affected by the tested concentrations of glycosides **1** and **2**, whereas cucumarioside A_0_-1 (positive control) significantly inhibited both PANC-1 and MCF-7 cell colonies at concentrations of 1 and 2 μM. Thus, compounds **1** and **2** exhibit a narrower spectrum of anticlonogenic action than the control, making them more selective toward TNBC cells. Additionally, the pronounced inhibitory effect of the glycosides against MDA-MB-468 cells makes this cell line a promising model for future investigation of the antitumor effects of glycosides.

## 3. Materials and Methods

### 3.1. General Experimental Procedures

The PerkinElmer 343 Polarimeter (PerkinElmer, Waltham, MA, USA) was used for specific rotation measurement. NMR spectra were registered on an Avance III 700 Bruker FT-NMR spectrometer (Bruker BioSpin GmbH, Rheinstetten, Germany) (700.13/176.04 MHz (^1^H/^13^C, 30 °C, *δ*_C_ 148.9 resonance of C_5_D_5_N/D_2_O for ^13^C and *δ*_H_ 7.21 resonance of C_5_D_5_N/D_2_O for ^1^H used as the references, BBO probe)). ESI MS (negative ion mode) spectra were obtained on an Agilent 6510 Q-TOF apparatus (Agilent Technology, Santa Clara, CA, USA), with a sample concentration of 0.01 mg/mL. HPLC was conducted on an Agilent 1260 Infinity II equipped with a differential refractometer (Agilent Technology, Santa Clara, CA, USA). The following columns were used: Supelco Discovery HS F5-5 (10 × 250 mm, 5 μM) (flow rate of 1.5 mL/min) and Diasfer C-8 (4.6 × 250 mm, 5 μM) (flow rate of 0.5 mL/min) (BioChemMac, Moscow, Russia).

### 3.2. Animals and Cells

Specimens of *Psolus phantapus* (family Psolidae; order Dendrochirotida) were collected from Avacha Bay by scuba diving. Sampling was performed in August 2014 at a depth of 5–15 m. Taxonomic identification of the animals was performed by Dr. Stepanov V.G. A voucher specimen is being kept in the Pacific Institute of Geography, Kamchatka Branch, Petropavlovsk-Kamchatsky, Russia.

Human erythrocytes were purchased from the Station of Blood Transfusion (Vladivostok, Russia). Human mammary epithelial cell line MCF-10A ^CRL-10317^; human breast cancer cell lines T-47D ^HTB-133^, MCF-7 ^HTB-22^, MDA-MB-468 ^HTB-132^, and MDA-MB-231 ^CRM-HTB-26^; and human pancreatic carcinoma PANC-1 ^CRL-1469^ were received from ATCC (Manassas, VA, USA).

The MCF-10 A cells were cultured in DMEM/F12 (Biolot, St. Petersburg, Russia) medium with 20% FBS (Biolot, St. Petersburg, Russia), 20 ng/mL of EGF (Sci-Store, Moscow, Russia) and 1% penicillin/streptomycin (Biolot, St. Petersburg, Russia).

The T-47D cells were cultured in RPMI-1640 medium (Biolot, St. Petersburg, Russia) with 10% FBS (Biolot, St. Petersburg, Russia) and 1% penicillin/streptomycin (Biolot, St. Petersburg, Russia).

The MCF-7, MDA-MB-468 and MDA-MB-231 cells were cultured in MEM medium (Biolot, St. Petersburg, Russia) with 10% FBS (Biolot, St. Petersburg, Russia) and 1% penicillin/streptomycin (Biolot, St. Petersburg, Russia).

The PANC-1 cells were cultured in DMEM (Biolot, St. Petersburg, Russia) with 10% FBS (Biolot, St. Petersburg, Russia) and 1% penicillin/streptomycin (Biolot, St. Petersburg, Russia).

All cell lines were incubated at 37 °C in an incubator with an atmosphere of 5% CO_2_.

### 3.3. Extraction and Isolation

Sea cucumbers (5 pieces) were minced and extracted twice with refluxing 60% EtOH (~4−5 h each time; the volume of the solvent is approximately equal to the volume occupied by animal material). After filtration, the extracts were evaporated in vacuo and chromatographed on a silica gel column with the solvent system CHCl_3_/EtOH (9/1) (V ~ 300 mL) to remove non-polar compounds (controlled with TLC). The remaining substances were eluted with the solvent system CHCl_3_/EtOH/H_2_O (10/15/4) (V ~ 400 mL), evaporated, then dissolved in H_2_O (V = 200 mL) and loaded on a Polychrom-1 column. The column was washed with H_2_O (V = 1000 mL) to remove salts and inorganic impurities. Crude glycosidic fraction (458 mg) was eluted with 50% EtOH (V = 350 mL). Then, it was subjected to twice-repeated silica gel column chromatography using solvent system CHCl_3_/EtOH/H_2_O (4:5:1) (V = 500 mL each), which resulted in isolation of one glycosidic subfraction (51.5 mg). The subsequent high-pressure liquid chromatography (HPLC) of this subfraction on a reversed-phase column Supelco Discovery HS F5-5 (10 × 250 mm) with MeOH/H_2_O/NH_4_OAc (1M water solution) in a ratio of (70/28/2) as the mobile phase yielded three fractions (fr. 1, Rt = 9–10 min; fr. 2, Rt = 12−13 min; and fr. 3, Rt = 14–14.5 min). Fractions 2 and 3 were re-chromatographed on a Diasfer C-8 (4.6 × 250 mm) column with solvent system MeOH/H_2_O/NH_4_OAc (1M water solution) in a ratio of (60/38/2) as the mobile phase resulted in the isolation of phantapusosides B (**2**) (1.0 mg, Rt = 13.5 min) and A (**1**) (1.0 mg, Rt = 15 min), correspondingly. Fraction 1 was subjected to HPLC on the Supelco Discovery HS F5-5 (10 × 250 mm) column with MeOH/H_2_O/NH_4_OAc (1M water solution) in a ratio of (60/38/2) as the mobile phase and gave psolusoside P (**3**) (1.5 mg, Rt = 16 min).

#### 3.3.1. *Phantapusoside A* (**1**)

Colorless powder; [α]_D_^20^-46° (*c* 0.1, H_2_O), mp 216 °C. Data of NMR: Table 1 and Table 2, Figures S1–S7. (*−*)HR-ESI-MS *m*/*z*: 1559.5249 (calc. 1559.5288) [M_2Na_ − Na]^−^, 768.2699 (calc. 768.2698) [M_2Na_ − Na]^2−^; (*−*)ESI-MS/MS *m*/*z*: 1439.5 [M_2Na_ − Na − NaHSO_4_]^−^, 1397.5 [M_2Na_ − Na − Glc (C_6_H_10_O_5_)]^−^, 1281.5 [M_2Na_ − Na − MeGlcSO_3_Na (C_7_H_11_O_8_SNa)]^−^, 1265.4 [M_2Na_ − Na − Glc (C_6_H_10_O_5_) − Xyl (C_5_H_8_O_4_)]^−^, 1119.4 [M_2Na_ − Na − Glc (C_6_H_10_O_5_)– Xyl (C_5_H_8_O_4_) − Qui (C_6_H_10_O_4_)]^−^, 999.4 [M_2Na_ − Na − Glc (C_6_H_10_O_5_)– Xyl (C_5_H_8_O_4_) − Qui (C_6_H_10_O_4_) − NaHSO_4_]^−^, 973.4 [M_2Na_ − Na − Agl (C_30_H_43_O_4_) − NaSO_4_]^−^, 667.0 [M_2Na_ − Na − Agl (C_30_H_43_O_3_) − Glc (C_6_H_10_O_5_) − Xyl (C_5_H_8_O_4_) − Qui (C_6_H_10_O_4_)– H]^−^.

#### 3.3.2. *Phantapusoside B* (**2**)

Colorless powder; [α]_D_^20^-38° (*c* 0.1, H_2_O), mp 204 °C. Data of NMR: Table 3 and Table S1, Figures S9–S15. (−)HR-ESI-MS *m*/*z*: 1559.5281 (calc. 1559.5288) [M_2Na_ − Na]^−^, 768.2702 (calc. 768.2698) [M_2Na_ − Na]^2−^; (*−*)ESI-MS/MS *m*/*z*: 1439.5 [M_2Na_ − Na − NaHSO_4_]^−^, 1281.5 [M_2Na_ − Na − MeGlcSO_3_Na (C_7_H_11_O_8_SNa)]^−^, 1119.4 [M_2Na_ − Na − Glc (C_6_H_10_O_5_)– Xyl (C_5_H_8_O_4_) − Qui (C_6_H_10_O_4_)]^−^, 667.0 [M_2Na_ − Na − Agl (C_30_H_43_O_3_) − Glc (C_6_H_10_O_5_) − Xyl (C_5_H_8_O_4_) − Qui (C_6_H_10_O_4_)– H]^−^; (*+*)ESI-MS/MS *m*/*z*: 1485 [M_2Na_ + Na − NaHSO_4_]^+^, 1327 [M_2Na_ + Na − MeGlcSO_3_ (C_7_H_11_O_8_SNa)]^+^, 1165 [M_2Na_ + Na − MeGlcSO_3_ (C_7_H_11_O_8_SNa) − Glc (C_6_H_10_O_5_)]^+^, 1045 [M_2Na_ + Na − MeGlcSO_3_ (C_7_H_12_O_9_SNa) − GlcSO_3_ (C_6_H_9_O_8_SNa) − H]^+^, 697 [M_2Na_ + Na − Agl (C_30_H_43_O_4_) − Glc (C_6_H_10_O_5_) − Xyl (C_5_H_8_O_4_) − Qui (C_6_H_10_O_4_) − H]^+^.

### 3.4. Cytotoxic Activity (MTT Assay)

The studied tumor cell lines (MCF-7, T-47D, MDA-MB-231, MDA-MB-468, and PANC-1), as well as a non-transformed human breast epithelial cell line (MCF-10A), were seeded in 96-well plates at a concentration of 6 × 10^3^ per well and allowed to adhere for 24 h. After the cells were plated, the studied glycosides were added to the plates at various concentrations (0.07–20 μM). Cucumarioside A_0_-1 (Cuc A_0_-1) was used at the same concentrations as a positive control. The cells were incubated for 24 h and then the medium was replaced with 100 μL of fresh medium, and 10 μL (5 mg/mL) of MTT (3-(4,5-dimethylthiazol-2-yl)-2,5-diphenyltetrazolium bromide) solution (neoFroxx, D-Einhausen, Germany) was added. The plates were incubated for 4 h and 100 μL of SDS-HCl solution (1 g of SDS/10 mL of d-H_2_O/17 μL of 6 N HCl) was added. The plates with cells were placed in a CO_2_ incubator overnight. The optical density was measured at a wavelength of 570 nm using a Synergy H1 microplate reader (BioTek Instruments Inc., Winooski, VT, USA). The cytotoxic activity of the studied glycosides was assessed by the IC_50_ value, the concentration at which the metabolic activity of cells is suppressed by 50%. The experiments were conducted in triplicate, *p* < 0.05.

### 3.5. Colony Formation Assay

A clonogenic assay was performed using the MDA-MB-231, MDA-MB-468, MCF-7, and PANC-1 cell lines. The cell lines were seeded in 6-well plates at a concentration of 0.3 × 10^2^/mL. Glycosides were added at concentrations of 0.2, 0.5, 1, and 2 μM 24 h after adhesion. The plates were left for 10–14 days in a CO_2_ incubator. Formed colonies (at least 50 cells) were fixed with methanol for 25 min and then stained with 0.5% crystal violet for 25 min. The plates containing the stained colonies were washed and air-dried. Colonies were quantified using the BIO-PRINT-Cx4 system (Vilber, Paris, France) in conjunction with Bio-Vision software (v. 18.01), following the manufacturer’s protocols. The data are expressed as the percentage of colony growth inhibition relative to the control group.

## Figures and Tables

**Table 1:** One- and two-dimensional NMR data of the aglycone moiety of phantapusoside A (**1**).

Position	*δ*_C,_ mult. ^a^	*δ*_H,_ mult. (*J* in Hz) ^b^	HMBC	ROESY
1	35.7, CH_2_	1.39, m		
		1.34, m		
2	26.8, CH_2_	2.04, m		
		1.84, m		H-19, H-30
3	89.1, CH	3.19, dd (11.8; 3.4)	C: 1 Xyl1	H-5, H-31, H-1 Xyl1
4	39.3, C			
5	48.2, CH	0.92, d (11.0; 4.2)	C: 19	H-3, H-31
6	23.1, CH_2_	1.93, m		
7	121.7, CH	5.66, m		H-15
8	143.9, C			
9	47.1, CH	3.54, brd (12.6)		H-19
10	35.5, C			
11	22.3, CH_2_	1.83, m		
		1.55, m		H-32
12	29.6, CH_2_	2.23, brd (11.0)		H-21
13	56.7, C			
14	45.6, C			
15	51.9, CH_2_	2.66, d (16.0)	C: 13, 16, 32	H-7, H-32
		2.33, d (16.0)	C: 14	
16	214.2, C			
17	63.4, CH	2.91, s	C: 12, 13, 16, 18, 20, 21	H-12, H-21, H-32
18	179.3, C			
19	23.9, CH_3_	1.13, s	C: 1, 5, 9, 10	H-1, H-2, H-9, H-30
20	83.8, C			
21	26.1, CH_3_	1.47, s	C: 17, 20, 22	H-12, H-17, H-22
22	38.2, CH_2_	1.70, m		
		1.55, m		
23	22.1, CH_2_	1.71, m		
		1.43, m		
24	37.8, CH_2_	1.88, m	C: 23, 25, 26	
25	145.5, C			
26	110.4, CH_2_	4.69, brs	C: 24, 27	H-27
		4.68, brs		H-27
27	22.1, CH_3_	1.62, s	C: 24, 25, 26	
30	17.2, CH_3_	1.02, s	C: 3, 4, 5, 31	H-2, H-6, H-31
31	28.6, CH_3_	1.17, s	C: 3, 4, 5, 30	H-3, H-5, H-6
32	31.8, CH_3_	1.17, s	C: 8, 13, 14, 15	H-11, H-15, H-17

^a^ Recorded at 176.04 MHz in C_5_D_5_N/D_2_O (4/1). ^b^ Recorded at 700.13 MHz in C_5_D_5_N/D_2_O (4/1). The original spectra of **1** are provided in Figures S1–S6.

**Table 2:** One- and two-dimensional NMR data of carbohydrate moiety of phantapusoside A (**1**).

Atom	δ_C_ mult. ^a,b,c^	δ_H_ mult. (*J* in Hz) ^d^	HMBC	ROESY
Xyl1 (1→C-3)				
1	104.8, CH	4.66, d (7.5)	C: 3	H-3; H-5 Xyl1
2	**82.0**, CH	3.98, t (8.6)	C: 1 Qui2; C: 1 Xyl1	H-1 Qui2
3	75.2, CH	4.18, t (8.6)		
4	**77.9**, CH	4.17, m		H-1 Glc5
5	63.6, CH_2_	4.39, dd (11.4; 5.1)		
		3.62, t (10.5)		
Qui2 (1→2Xyl1)				
1	104.5, CH	5.07, d (8.0)	C: 2 Xyl1	H-2 Xyl1; H-3, 5 Qui2
2	75.8, CH	3.87, t (9.0)	C: 1 Qui2	H-4 Qui2
3	75.1, CH	3.99, t (9.0)	C: 4 Qui2	
4	**85.7**, CH	3.48, t (9.0)	C: 1 Xyl3	H-1 Xyl3; H-2 Qui2
5	71.4, CH	3.69, dd (9.0; 6.0)		H-1 Qui2
6	17.8, CH_3_	1.60, d (6.0)	C: 4, 5 Qui2	H-4 Qui2
Xyl3 (1→4Qui2)				
1	104.5, CH	4.75, d (8.1)	C: 4 Qui2	H-4 Qui2; H-3, 5 Xyl3
2	73.4, CH	3.88, t (9.1)	C: 1, 3 Xyl3	
3	**86.3**, CH	4.12, t (9.1)	C: 2, 4 Xyl3, C: 1 Glc4	H-1 Glc4; H-1 Xyl3
4	68.8, CH	3.94, m		
5	65.9, CH_2_	4.12, m		H-1 Xyl3
		3.60, t (11.1)		H-1 Xyl3
Glc4 (1→3Xyl3)				
1	104.6, CH	5.22, d (7.9)	C: 3 Xyl3	H-3 Xyl3; H-3, 5 Glc4
2	74.8, CH	3.95, t (9.2)	C: 1, 3 Glc4	
3	77.2, CH	4.13, t (9.2)	C: 4 Glc4	
4	71.1, CH	3.92, m	C: 3, 5 Glc4	
5	77.7, CH	3.92, m		H-1, 3 Glc4
6	62.0, CH_2_	4.40, d (12.6)		
		4.05, dd (12.6; 5.4)		
Glc5 (1→4Xyl1)				
1	102.2, CH	4.90, d (7.5)	C: 4 Xyl1	H-4 Xyl1; H-3, 5 Glc5
2	73.2, CH	3.83, t (9.5)	C: 1 Glc5	
3	**86.1**, CH	4.14, t (9.5)	C: 1 MeGlc6; C: 2, 4 Glc5	H-1 MeGlc6; H-1 Glc5
4	69.0, CH	3.86, t (9.5)		
5	74.9, CH	4.06, m		H-1 Glc5
6	*67.3*, CH_2_	4.98, brd (9.4)		
		4.68, brd (9.4)		
MeGlc6 (1→3Glc51)				
1	104.4, CH	5.15, d (7.7)	C: 3 Glc5	H-3 Glc5; H-3,5 MeGlc6
2	74.2, CH	3.78, t (9.5)	C: 1,3 MeGlc6	
3	86.4, CH	3.63, t (9.5)	C: 2, 4 MeGlc6; OMe	H-1 MeGlc6
4	69.7, CH	4.04, t (9.5)	C: 5 MeGlc6	
5	75.6, CH	3.99, m		H-1 MeGlc6
6	*66.9*, CH_2_	4.93, d (10.7)		
		4.78, dd (12.6; 5.8)		
OMe	60.5, CH_3_	3.75, s	C: 3 MeGlc6	

^a^ Recorded at 176.04 MHz in C_5_D_5_N/D_2_O (4/1). ^b^ Bold = interglycosidic positions. ^c^ Italic = sulfate positions. ^d^ Recorded at 700.13 MHz in C_5_D_5_N/D_2_O (4/1). Multiplicity by 1D TOCSY. The original spectra of **1** are provided in Figures S1–S7.

**Table 3:** One- and two-dimensional NMR data of carbohydrate moiety of phantapusoside B (**2**).

Atom	δ_C_ mult. ^a,b,c^	δ_H_ mult. (*J* in Hz) ^d^	HMBC	ROESY
Xyl1 (1→C-3)				
1	104.8, CH	4.66, d (7.2)	C: 3	H-3; H-3, 5 Xyl1
2	**82.0**, CH	3.97, t (8.0)	C: 1 Qui2; C: 3 Xyl1	H-1 Qui2
3	75.2, CH	4.17, m	C: 4 Xyl1	
4	**77.9**, CH	4.17, m		
5	63.6, CH_2_	4.38, brd (10.9)		
		3.62, t (9.4)		H-1 Xyl1
Qui2 (1→2Xyl1)				
1	104.4, CH	5.07, d (7.8)	C: 2 Xyl1	H-2 Xyl1; H-5 Qui2
2	75.8, CH	3.86, t (9.3)	C: 1 Qui2	
3	74.8, CH	3.99, t (9.3)	C: 4 Qui2	
4	**85.7**, CH	3.48, t (9.3)	C: 1 Xyl3; C: 3, 5 Qui2	H-1 Xyl3
5	71.4, CH	3.69 dd, (9.3; 5.4)		H-1 Qui2
6	17.8, CH_3_	1.59, d (5.4)	C: 4, 5 Qui2	
Xyl3 (1→4Qui2)				
1	104.4, CH	4.75, d (7.1)	C: 4 Qui2	H-4 Qui2; H-3, 5 Xyl3
2	73.4, CH	3.88, t (8.4)	C: 1 Xyl3	
3	**86.4**, CH	4.10, t (8.4)	C: 2 Xyl3, C: 1 Glc4	H-1 Glc4; H-1, 5 Xyl3
4	68.8, CH	3.93, m		
5	65.9, CH_2_	4.12, dd (11.6; 5.2)	C: 3, 4 Xyl3	
		3.59 t (11.0)	C: 1, 3, 4 Xyl3	H-1 Xyl3
Glc4 (1→3Xyl3)				
1	104.6, CH	5.19, d (7.3)	C: 3 Xyl3	H-3 Xyl3; H-3, 5 Glc4
2	74.9, CH	3.94, t (8.1)	C: 1, 3 Glc4	
3	77.2, CH	4.11, t (8.1)	C: 4 Glc4	
4	71.1, CH	3.90, m	C: 5 Glc4	
5	77.7, CH	3.90, m	C: 6 Glc4	H-1 Glc4
6	62.0, CH_2_	4.39, d (11.1)		
		4.05, dd (11.1; 4.2)	C: 5 Glc4	
Glc5 (1→4Xyl1)				
1	102.2, CH	4.90, d (8.6)	C: 4 Xyl1	H-4 Xyl1; H-3, 5 Glc5
2	73.4, CH	3.84, t (9.4)	C: 1, 3 Glc5	
3	**86.0**, CH	4.16, t (9.4)	C: 1 MeGlc6; C: 2, 4 Glc5	H-1 MeGlc6; H-1 Glc5
4	68.9, CH	3.86, t (9.4)	C: 5, 6 Glc5	
5	74.9, CH	4.04, m		H-1 Glc5
6	*67.2*, CH_2_	4.95, brd (10.9)		
		4.67, m		
MeGlc6 (1→3Glc51)				
1	104.4, CH	5.17, d (7.9)	C: 3 Glc5	H-3 Glc5; H-3,5 MeGlc6
2	74.0, CH	3.86, t (9.4)	C: 1,3 MeGlc6	H-4 MeGlc5
3	85.3, CH	3.72, t (9.4)	C: 2, 4 MeGlc6; OMe	H-1 MeGlc6
4	*76.2*, CH	4.89, t (9.4)	C: 3, 5, 6 MeGlc6	H-2 MeGlc6
5	76.5, CH	3.84, m		H-1 MeGlc6
6	61.8, CH_2_	4.49, d (11.6)		
		4.34, d (12.3; 5.1)		
OMe	60.7, CH_3_	3.93, s	C: 3 MeGlc6	

^a^ Recorded at 176.04 MHz in C_5_D_5_N/D_2_O (4/1). ^b^ Bold = interglycosidic positions. ^c^ Italic = sulfate positions. ^d^ Recorded at 700.13 MHz in C_5_D_5_N/D_2_O (4/1). Multiplicity by 1D TOCSY. The original spectra of **1** are provided in Figures S9–S15.

**Table 4:** The cytotoxic activities of glycosides **1** and **2** and cucumarioside A_0_-1 (positive control) against human erythrocytes and MCF-10A, MCF-7, T-47D, MDA-MB-231, MDA-MB-468, and PANC-1 human cell lines.

Glycosides	ED_50_, µM, Erythrocytes	Cytotoxicity, IC_50_ µM					
		MCF-10A	MCF-7	T-47D	MDA-MB-231	MDA-MB-468	PANC-1
phantapusoside A (**1**)	0.48 ± 0.02	>20.0	15.33 ± 0.57	>20.0	14.41 ± 0.65	13.82 ± 0.31	13.38 ± 0.33
phantapusoside B (**2**)	0.47 ± 0.02	14.01 ± 0.62	12.71 ± 0.81	17.10 ± 0.27	9.52 ± 0.45	7.46 ± 0.33	7.68 ± 0.26
cucumarioside A_0_-1	1.05 ± 0.15	12.48 ± 0.87	13.30 ± 1.26	14.16 ± 1.53	5.58 ± 0.78	5.02 ± 0.54	5.23 ± 0.48

**Table 5:** The inhibition of colony formation of MDA-MB-231, MDA-MB-468, MCF-7, and PANC-1 human cell lines by the glycosides **1** and **2** and cucumarioside A_0_-1 (positive control).

Glycosides	Colony Formation, IC_50_, µM			
	MDA-MB-231	MDA-MB-468	MCF-7	PANC-1
phantapusoside A (**1**)	1.12	0.41	1.87	>2.0
phantapusoside B (**2**)	0.54	<0.2	>2.0	>2.0
cucumarioside A_0_-1	0.29	0.32	0.60	0.40

## Data Availability

The original contributions presented in this study are included in the article. Further inquiries can be directed to the corresponding author.

## Supplementary Materials

The following supporting information can be downloaded at https://www.mdpi.com/article/10.3390/md24060202/s1, Figures S1–S18: The original spectral data of compounds **1**–**3**; Table S1: One and two-dimensional NMR data of aglycone of phantapusoside B (**2**).
