# Supplementary material for: Three Sulfated Triterpene Glycosides from the Sea Cucumber Psolus phantapus—Biological Activity Against Human Cancer Cell Lines
Source: Mar Drugs. 2026 Jun 8;24(6):202. doi: 10.3390/md24060202 (PMC13301897; doi:10.3390/md24060202)
Supplement: Supplementary file 1 [file marinedrugs-24-00202-s001.zip › marinedrugs-4345002-supplementary.pdf]

Supplementary data content page

Title: Three Sulfated Triterpene Glycosides from the Sea Cucumber Psolus phantapus—Biological Activity against Human Cancer Cell lines

Authors: Alexandra S. Silchenko [1,*], Ekaterina A. Chingizova [1], Ekaterina S. Menchinskaya [1], Kseniya M. Tabakmakher [1], Anatoly I. Kalinovsky [1], Sergey A. Avilov [1], Roman S. Popov [1], Vadim G. Stepanov [2] and Vladimir I. Kalinin [1]

Address: [1]*G.B. Elyakov Pacific Institute of Bioorganic Chemistry, Far Eastern Branch of the Russian Academy of Sciences, Pr. 100-letya Vladivostoka 159, 690022 Vladivostok, Russia*

[2]*Kamchatka Branch of Pacific Institute of Geography, Far Eastern Branch of the Russian Academy of Sciences, Partizanskaya St. 6, 683000 Petropavlovsk-Kamchatsky, Russia; stepanovvadim24@gmail.com (V.G.S.)*

Correspondence: silchenko_als@piboc.dvo.ru; Tel.: +7-423-231-1168

Content:

Figure S1. The ^{13}C NMR (176.04 MHz) spectrum of phantapusoside A (**1**) in C_5D_5N/D_2O (4/1)

Figure S2. The ^{1}H NMR (700.13 MHz) spectrum of phantapusoside A (**1**) in C_5D_5N/D_2O (4/1)

Figure S3. The COSY (700.13 MHz) spectrum of phantapusoside A (**1**) in C_5D_5N/D_2O (4/1)

Figure S4. The HSQC (700.13 MHz) spectrum of phantapusoside A (**1**) in C_5D_5N/D_2O (4/1)

Figure S5. The ROESY (700.13 MHz) spectrum of phantapusoside A (**1**) in C_5D_5N/D_2O (4/1)

Figure S6. The HMBC (700.13 MHz) spectrum of phantapusoside A (**1**) in C_5D_5N/D_2O (4/1)

Figure S7. 1 D TOCSY (700.13 MHz) spectra of Xyl1, Qui2, Xyl3, Glc4, Glc5 and MeGlc6 of phantapusoside A (**1**) in C_5D_5N/D_2O (4/1)

Figure S8. (–)HR-ESI-MS and (–)HR-ESI-MS/MS spectra of phantapusoside A (**1**)

Figure S9. The ^{13}C NMR (176.04 MHz) spectrum of phantapusoside B (**2**) in C_5D_5N/D_2O (4/1)

Figure S10. The ^{1}H NMR (700.13 MHz) spectrum of phantapusoside B (**2**) in C_5D_5N/D_2O (4/1)

Figure S11. The COSY (700.13 MHz) spectrum of phantapusoside B (**2**) in C_5D_5N/D_2O (4/1)

Figure S12. The HSQC (700.13 MHz) spectrum of phantapusoside B (**2**) in C_5D_5N/D_2O (4/1)

Figure S13. The ROESY (700.13 MHz) spectrum of phantapusoside B (**2**) in C_5D_5N/D_2O (4/1)

Figure S14. The HMBC (700.13 MHz) spectrum of phantapusoside B (**2**) in C_5D_5N/D_2O (4/1)

Figure S15. 1 D TOCSY (700.13 MHz) spectra of Xyl1, Qui2, Xyl3, Glc4, Glc5 and MeGlc5 of phantapusoside B (**2**) in C_5D_5N/D_2O (4/1)

Figure S16. (–)HR-ESI-MS and HR-ESI-MS/MS spectra of phantapusoside B (**2**)

Figure S17. The ^{13}C NMR (176.04 MHz) spectrum of psolusoside P (**3**) in C_5D_5N/D_2O (4/1)

Figure S18. HR-ESI-MS(–) spectrum of psolusoside P (**3**)

Table S1. One- and two-dimensional NMR data of aglycone moiety of phantapusoside B (**2**)

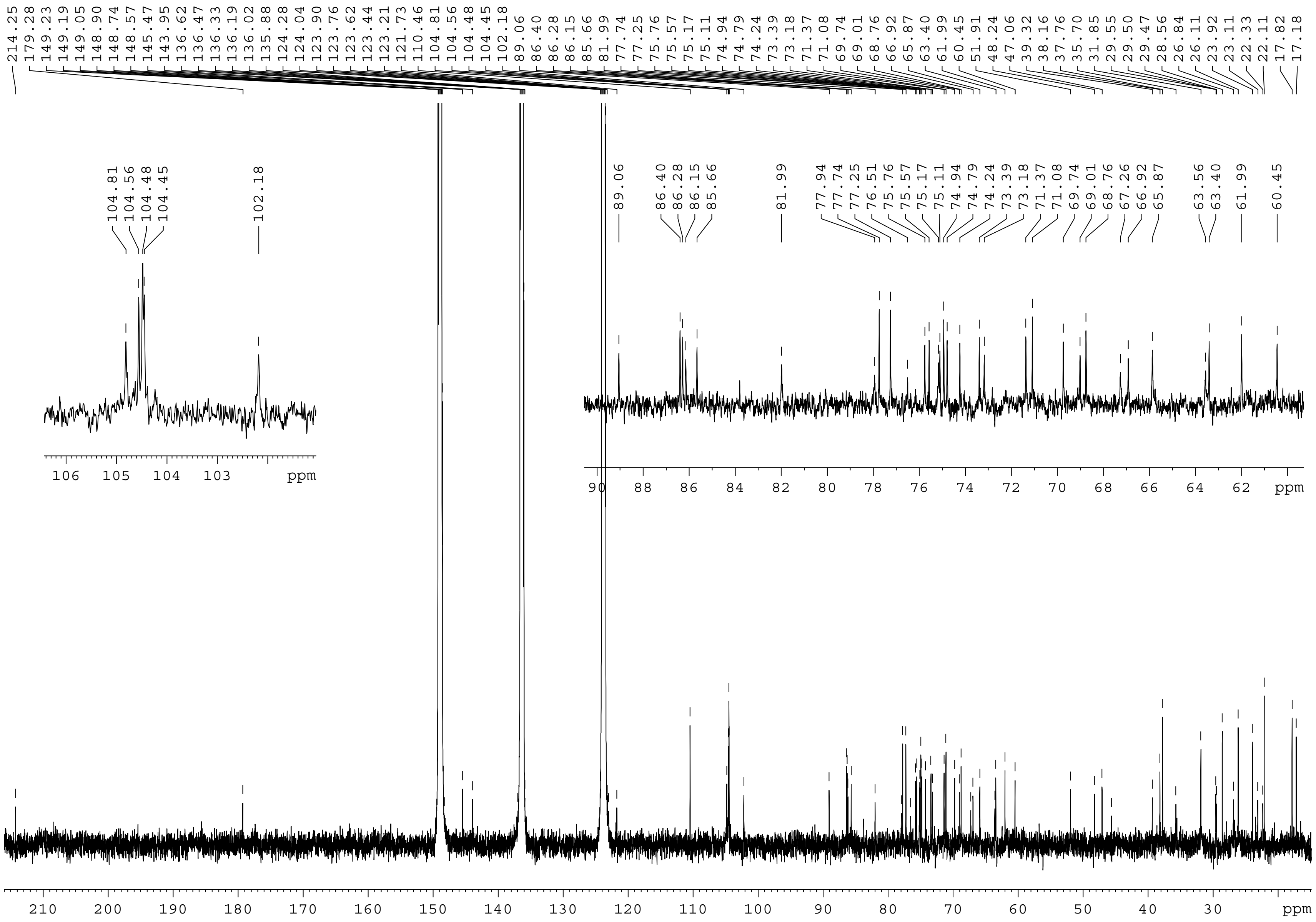

Figure S1. The ^{13}C NMR (176.04 MHz) spectrum of phantapusoside A (**1**) in C_5D_5N/D_2O (4/1)

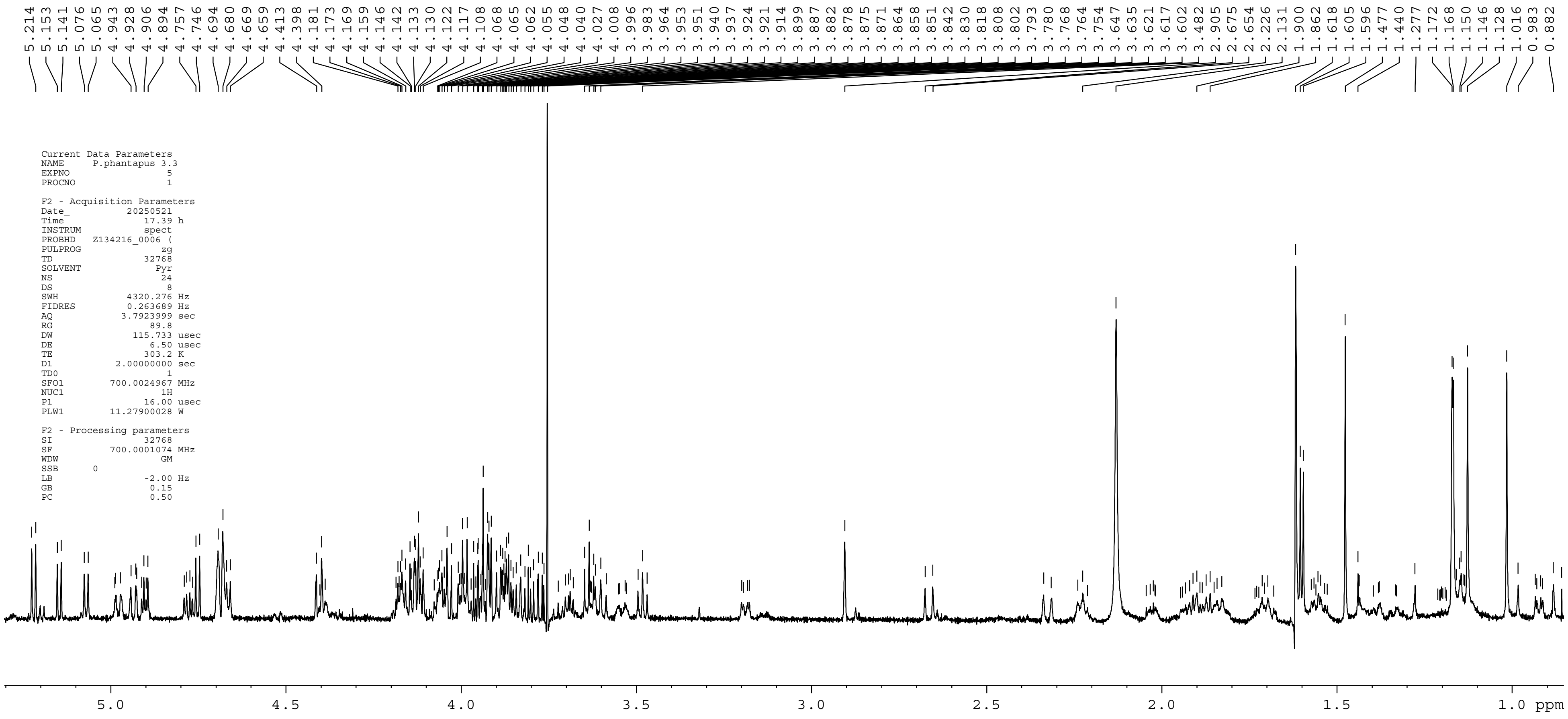

Figure S2. The ^{1}H NMR (700.13 MHz) spectrum of phantapusoside A (**1**) in C_5D_5N/D_2O (4/1)

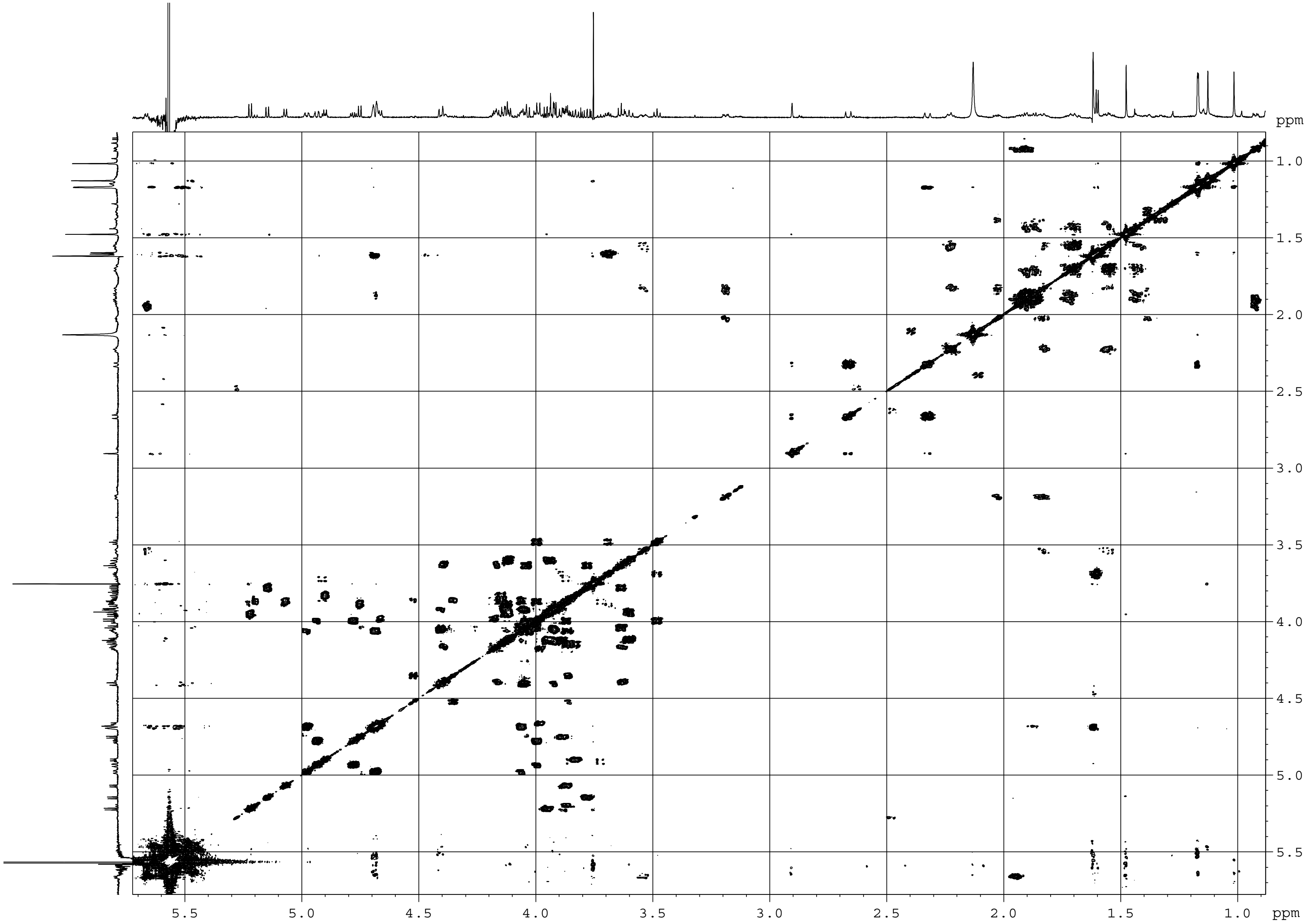

Figure S3. The COSY (700.13 MHz) spectrum of phantapusoside A (**1**) in C_5D_5N/D_2O (4/1)

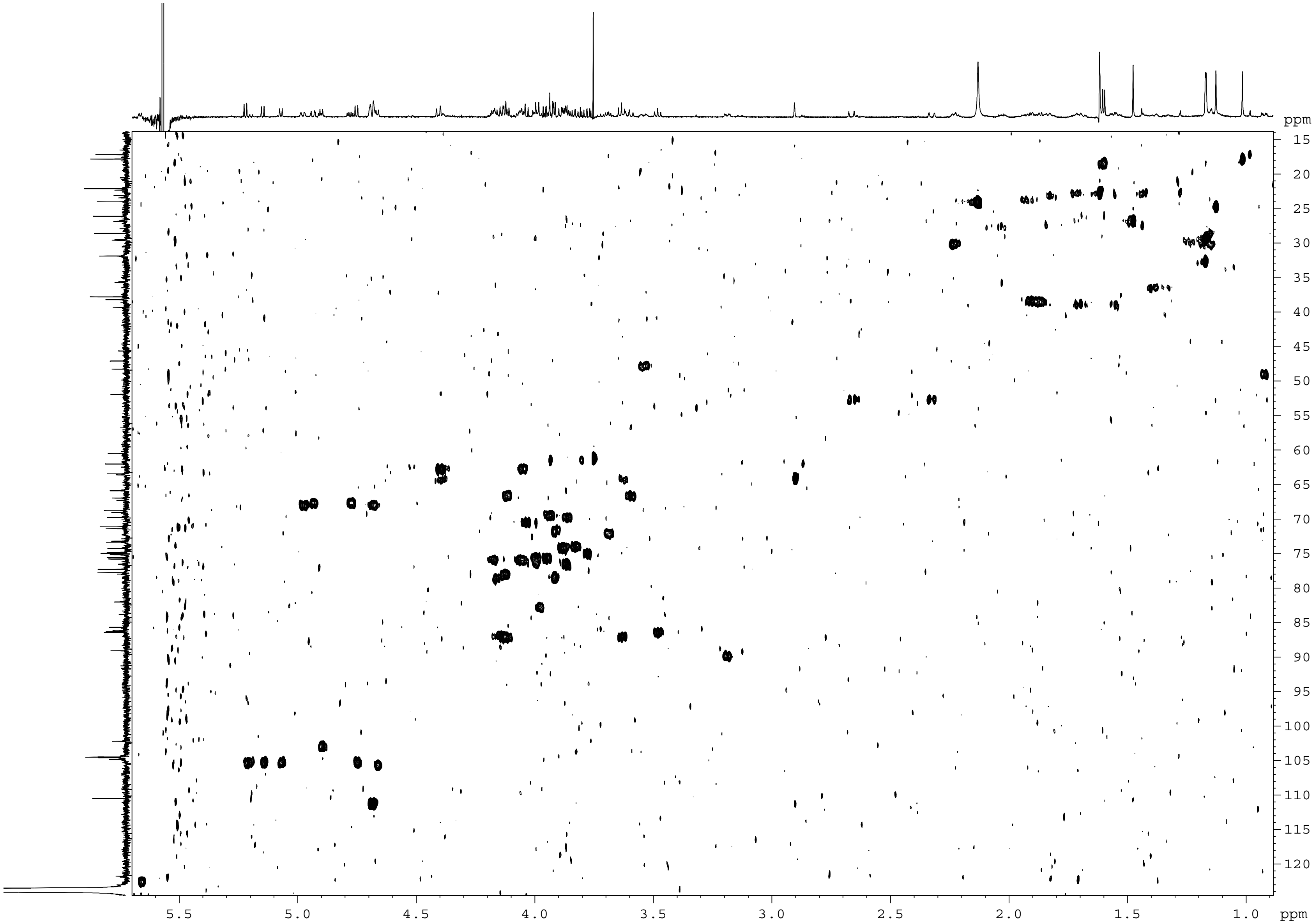

Figure S4. The HSQC (700.13 MHz) spectrum of phantapusoside A (**1**) in C_5D_5N/D_2O (4/1)

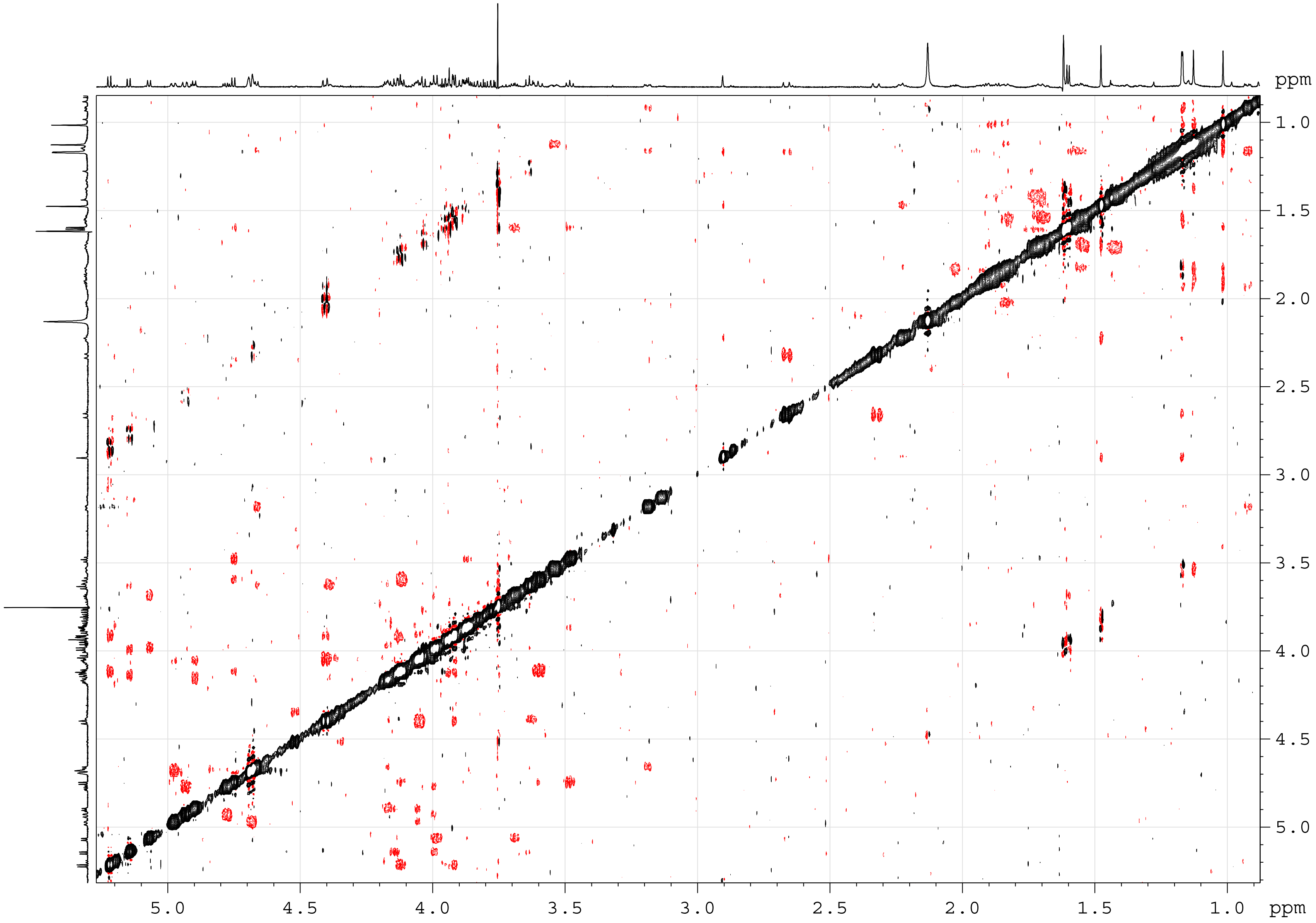

Figure S5. The ROESY (700.13 MHz) spectrum of phantapusoside A (**1**) in C_5D_5N/D_2O (4/1)

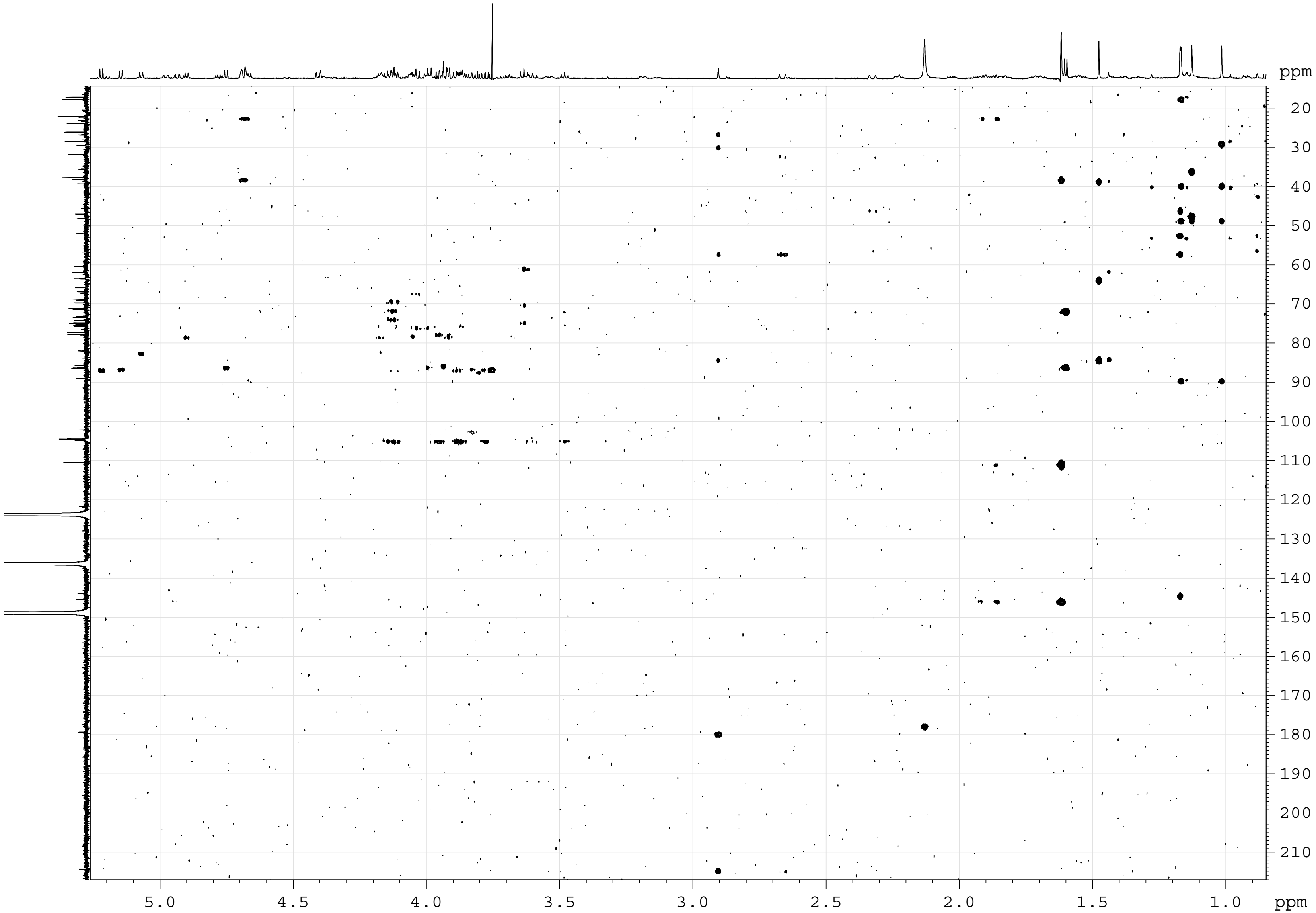

Figure S6. The HMBC (700.13 MHz) spectrum of phantapusoside A (**1**) in C_5D_5N/D_2O (4/1)

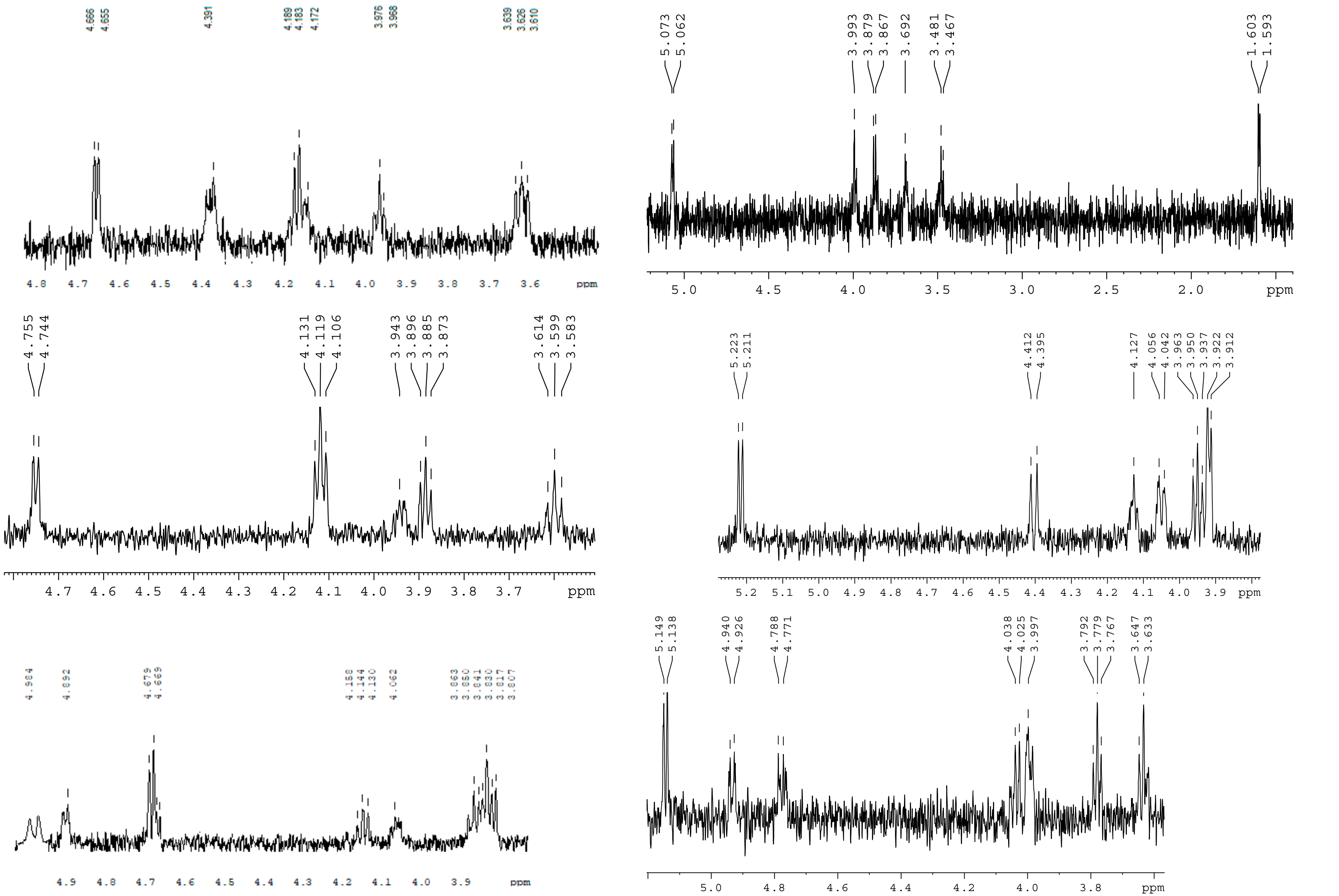

Figure S7. 1 D TOCSY (700.13 MHz) spectra of Xyl1, Qui2, Xyl3, Glc4, Glc5 and MeGlc6 of phantapusoside A (**1**) in C_5D_5N/D_2O (4/1)

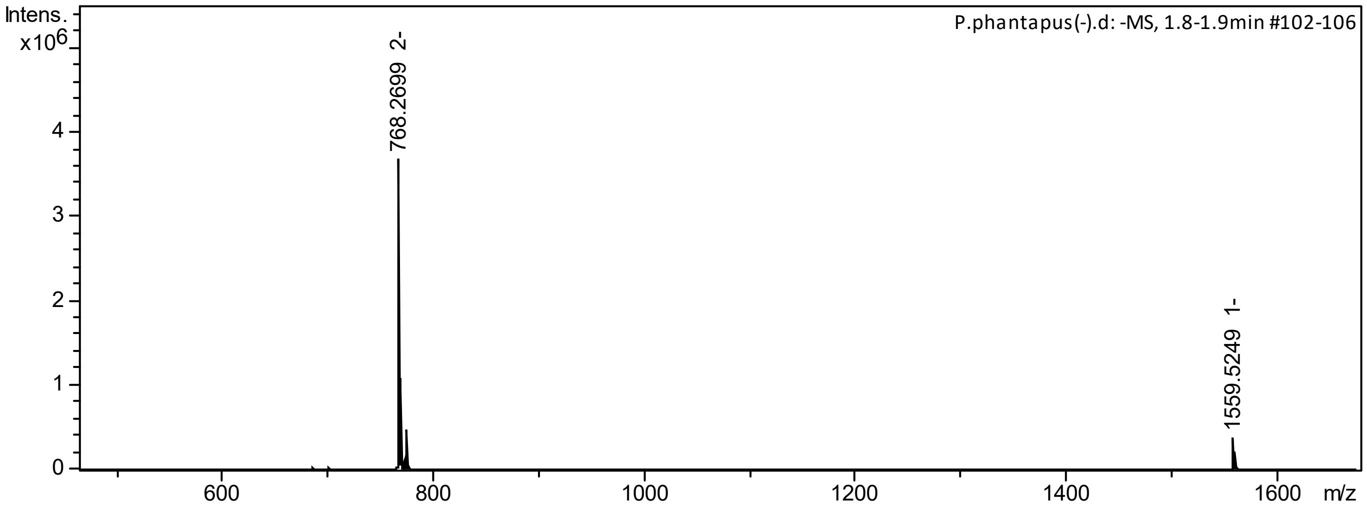

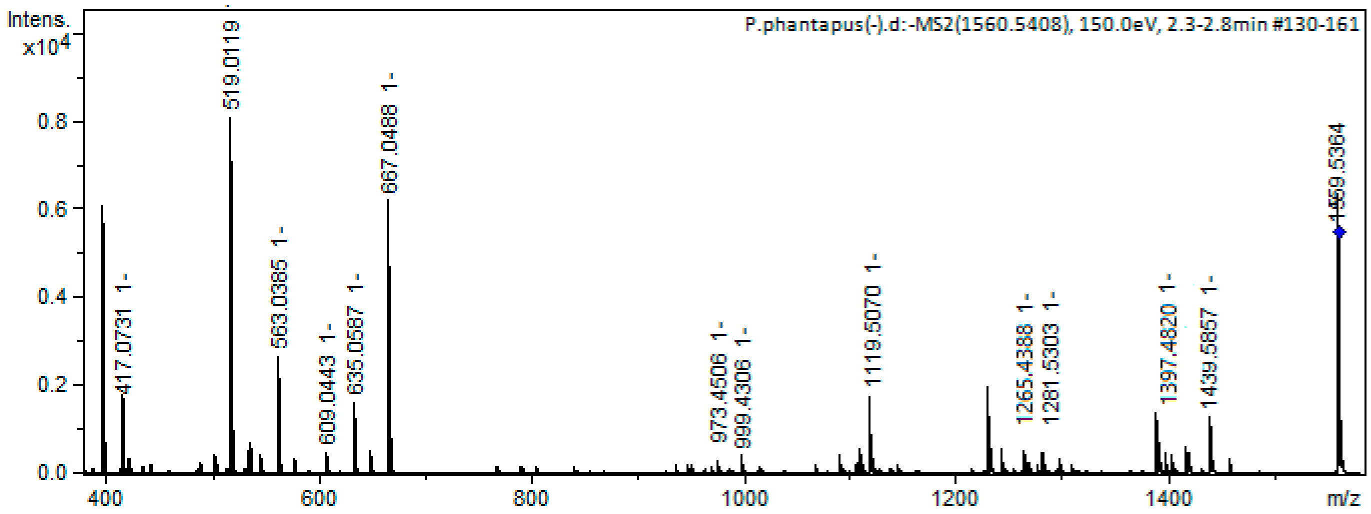

Figure S8. (–)HR-ESI-MS and (–)ESI-MS/MS spectra of phantapusoside A (**1**)

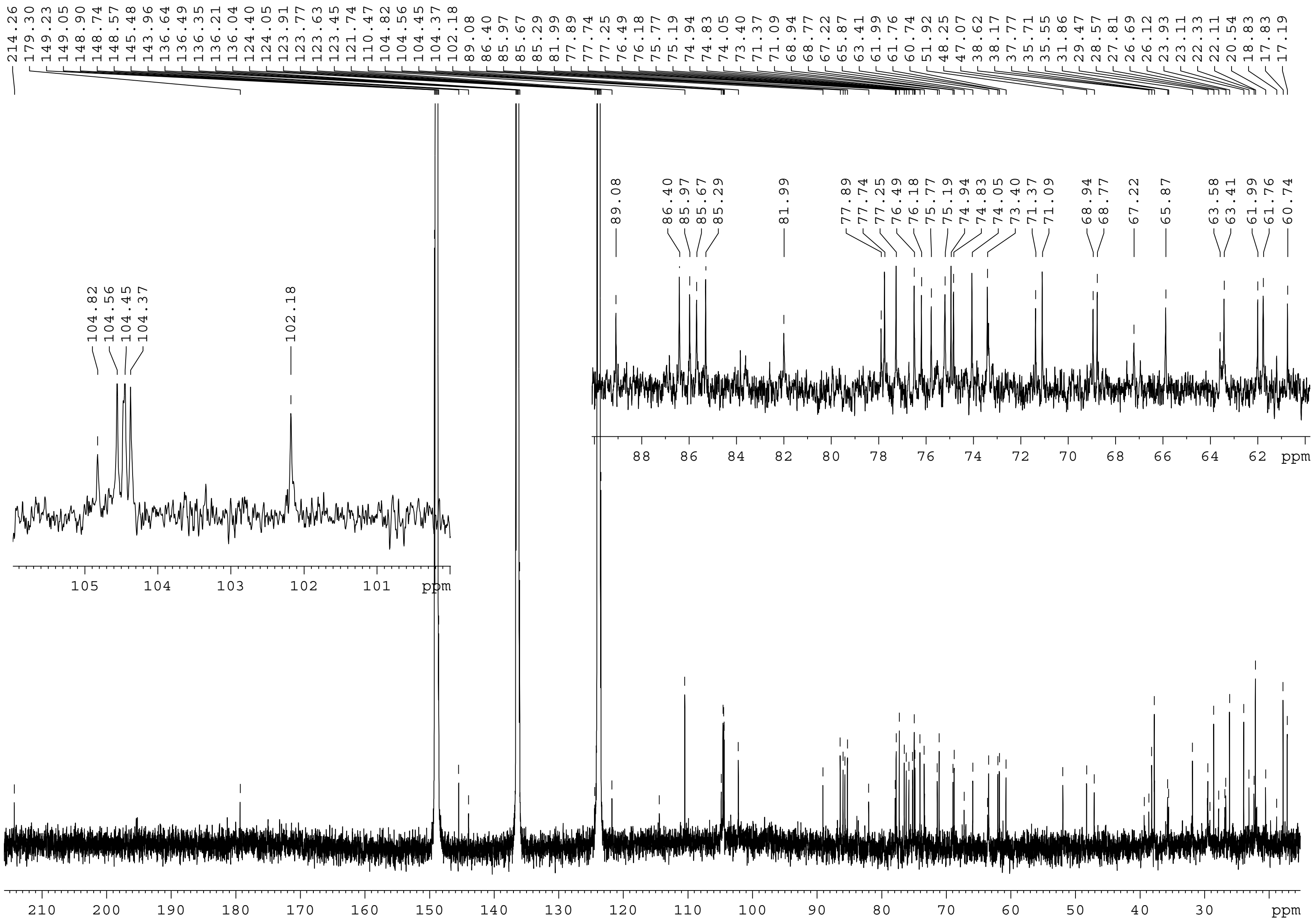

Figure S9. The ^{13}C NMR (176.04 MHz) spectrum of phantapusoside B (**2**) in C_5D_5N/D_2O (4/1)

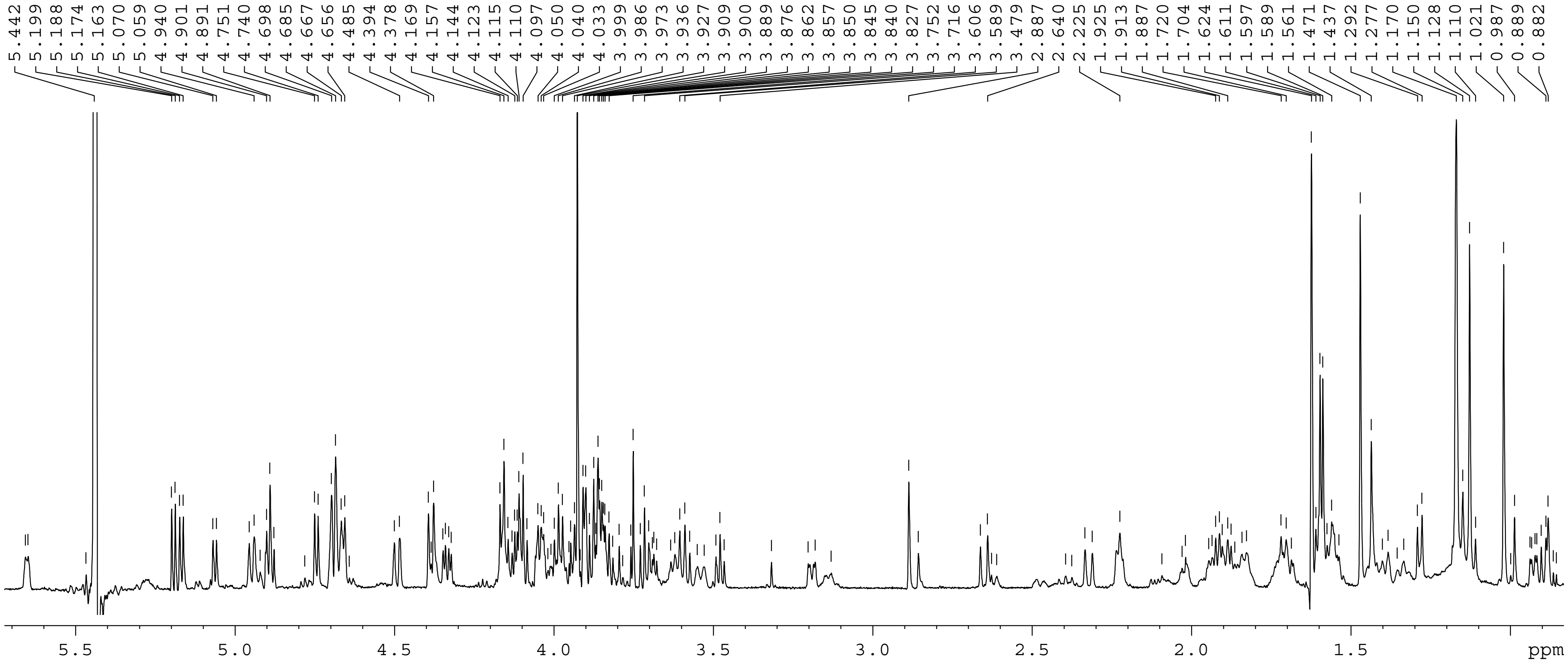

Figure S10. The ^{1}H NMR (700.13 MHz) spectrum of phantapusoside B (**2**) in C_5D_5N/D_2O (4/1)

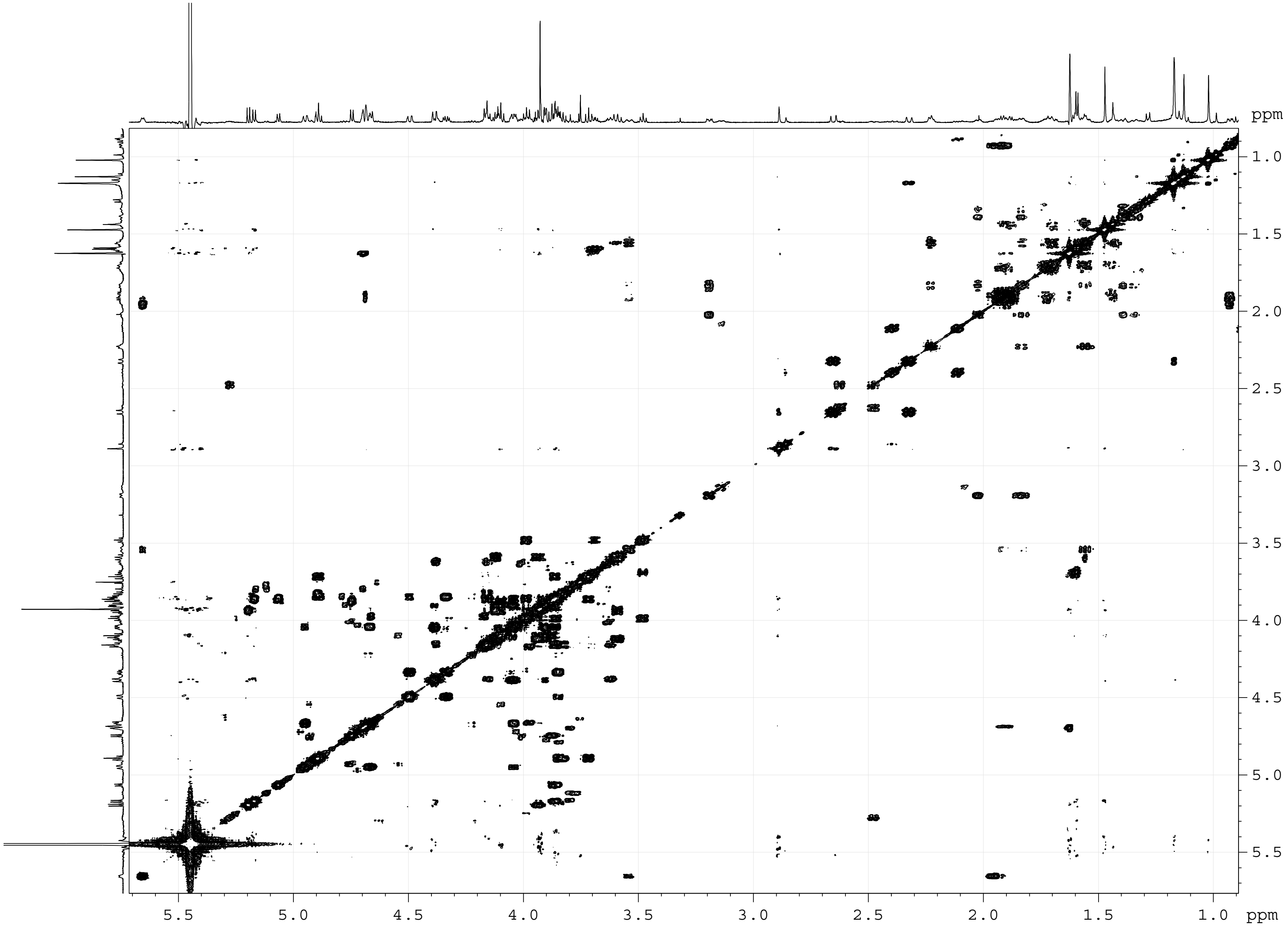

Figure S11. The COSY (700.13 MHz) spectrum of phantapusoside B (**2**) in C_5D_5N/D_2O (4/1)

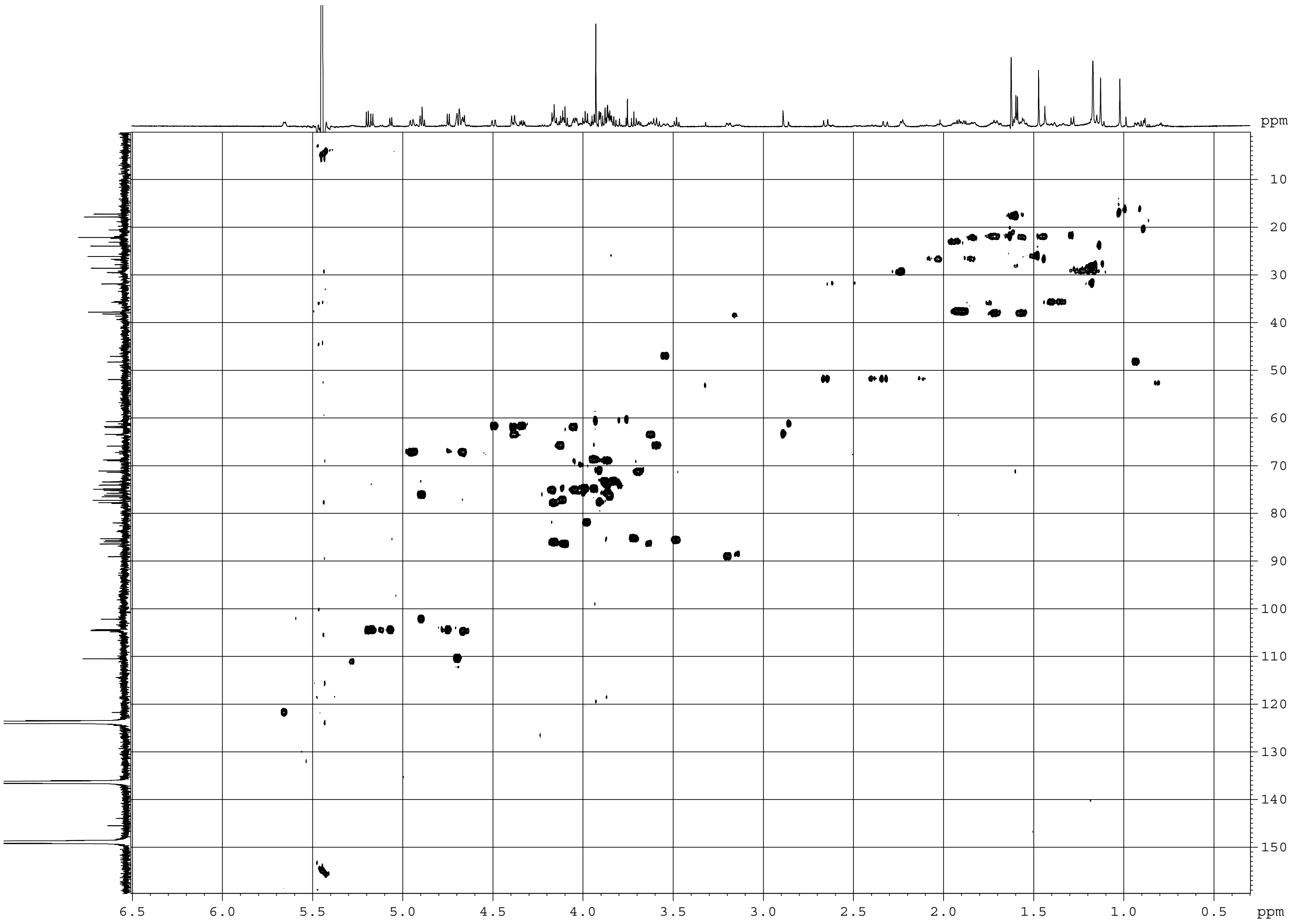

Figure S12. The HSQC (700.13 MHz) spectrum of phantapusoside B (**2**) in C_5D_5N/D_2O (4/1)

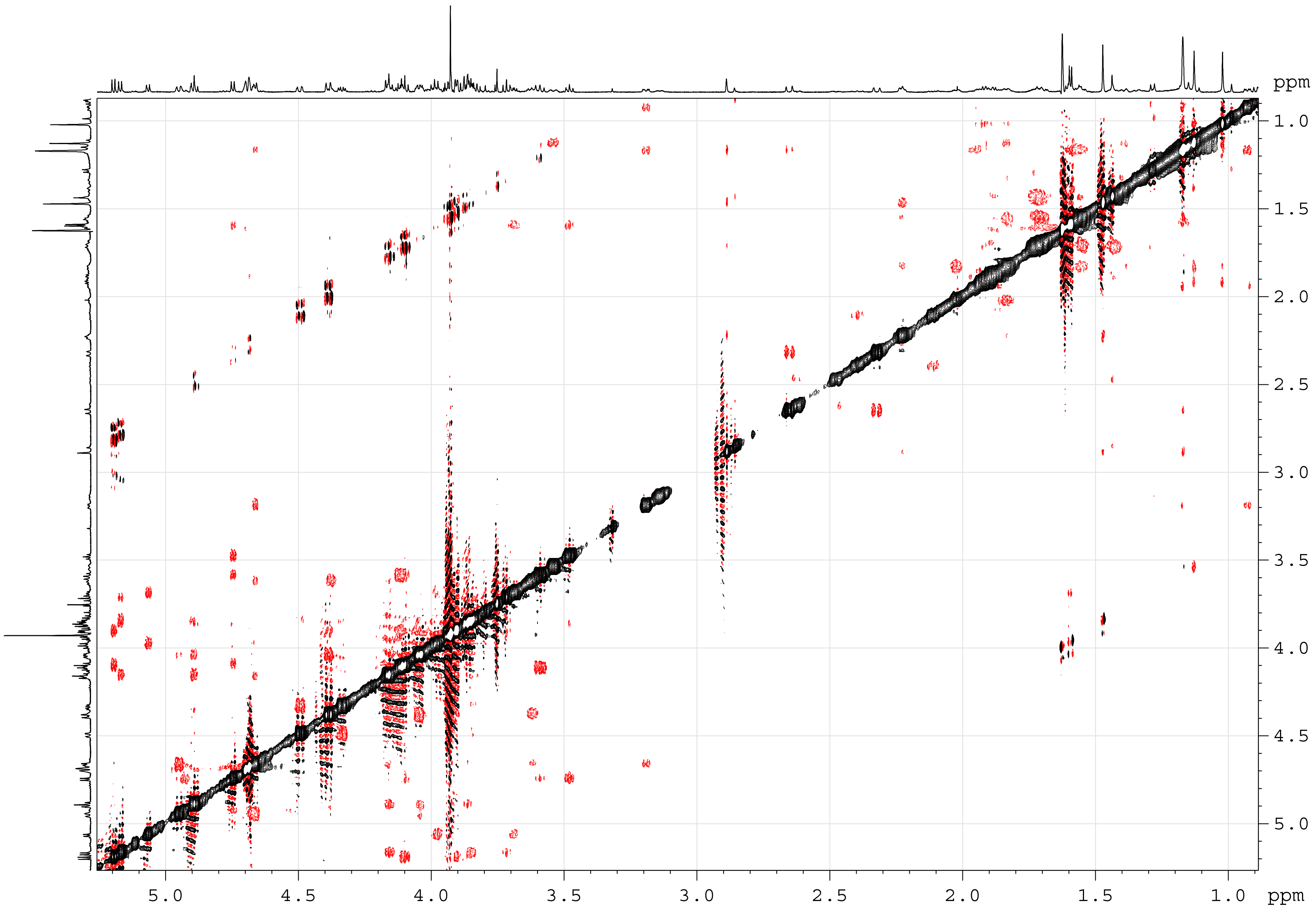

Figure S13. The ROESY (700.13 MHz) spectrum of phantapusoside B (**2**) in C_5D_5N/D_2O (4/1)

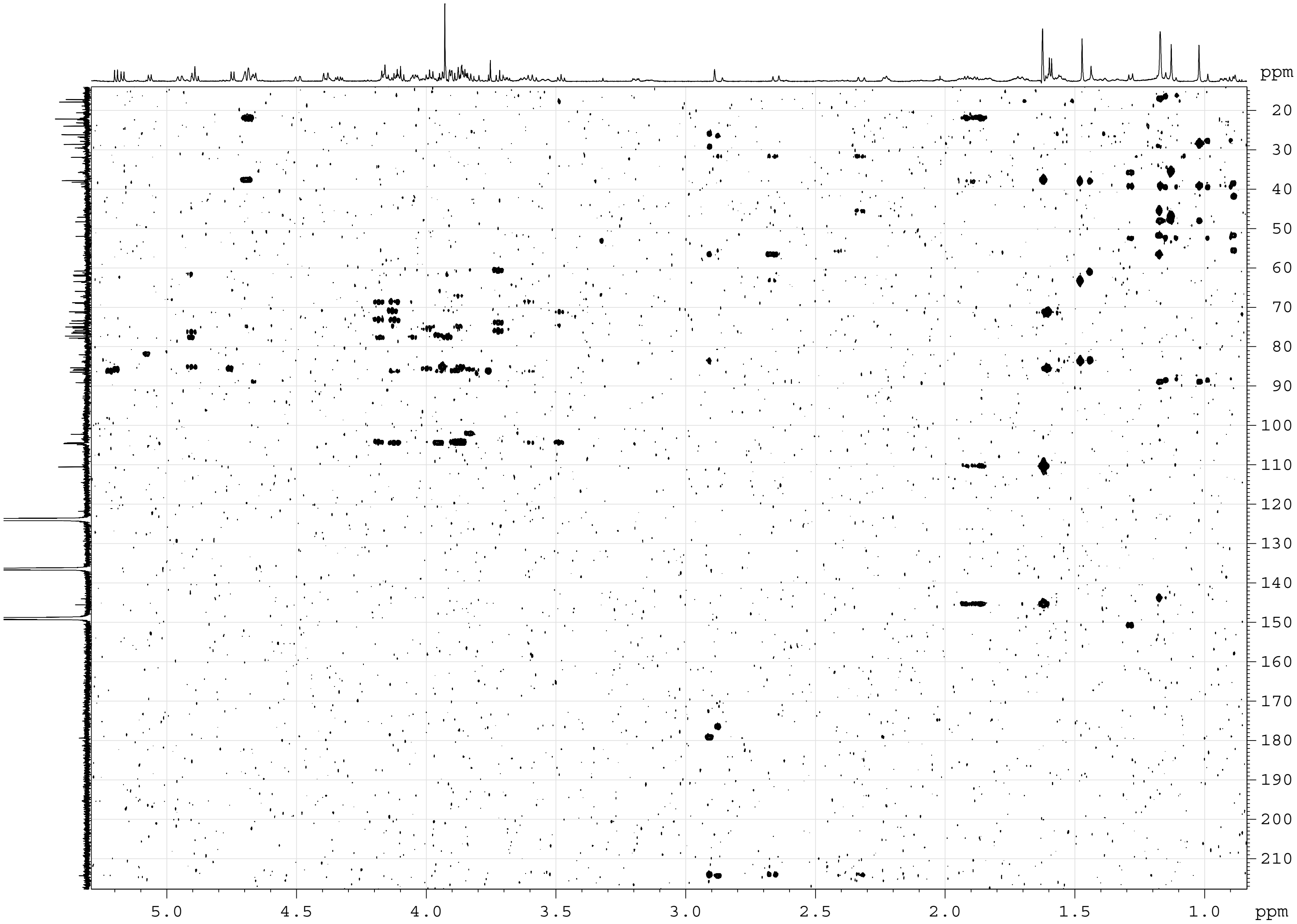

Figure S14. The HMBC (700.13 MHz) spectrum of phantapusoside B (**2**) in C_5D_5N/D_2O (4/1)

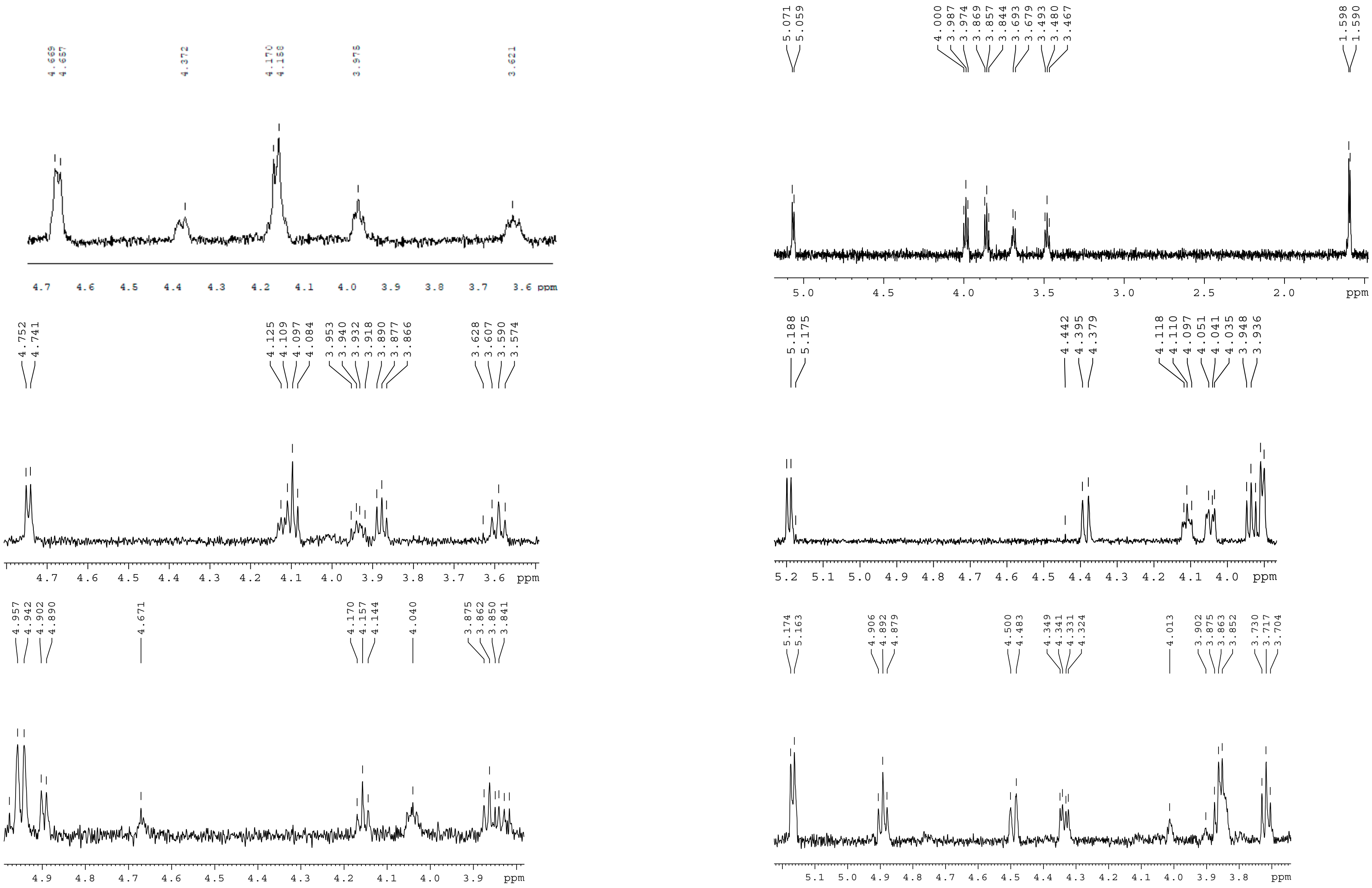

Figure S15. 1 D TOCSY (700.13 MHz) spectra of Xyl1, Qui2, Xyl3, Glc4, Glc5 and MeGlc5 of phantapusoside B (**2**) in C_5D_5N/D_2O (4/1)

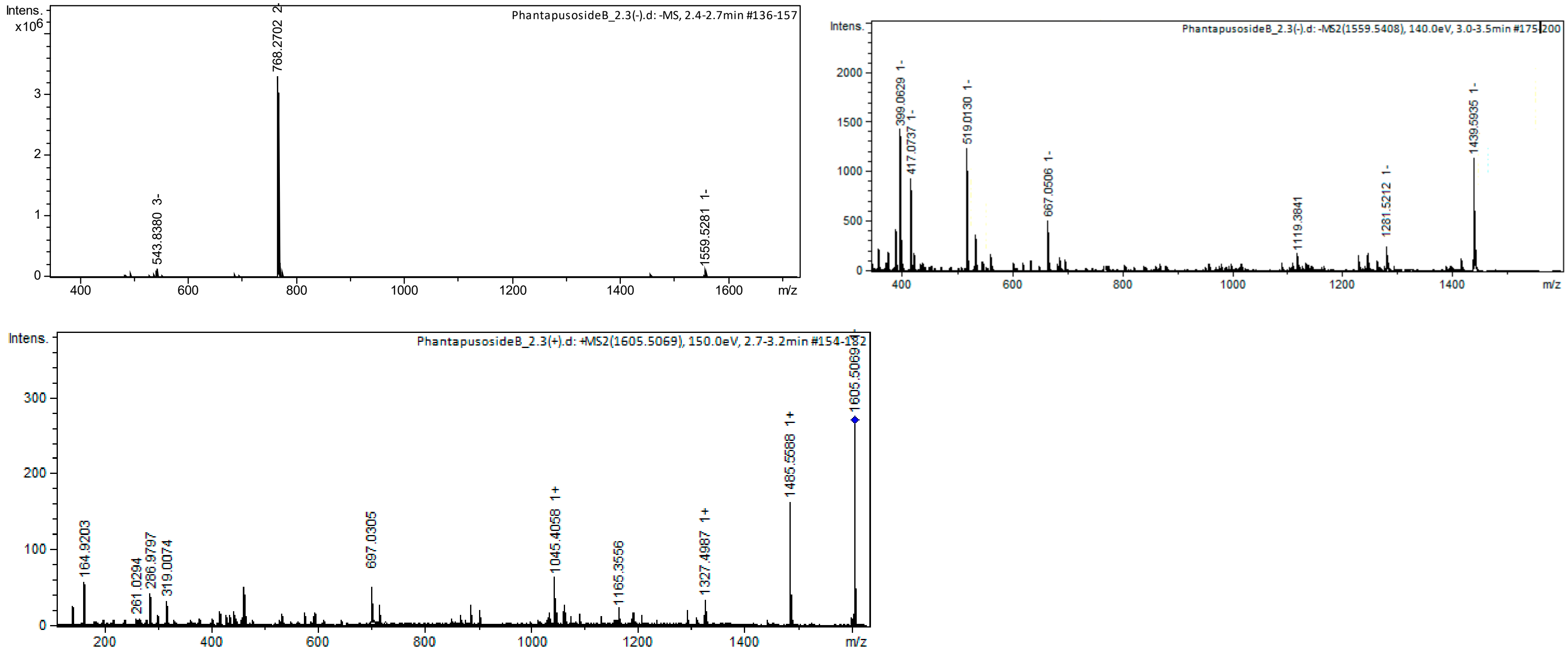

Figure S16. (–)HR-ESI-MS and ESI-MS/MS spectra of phantapusoside B (**2**)

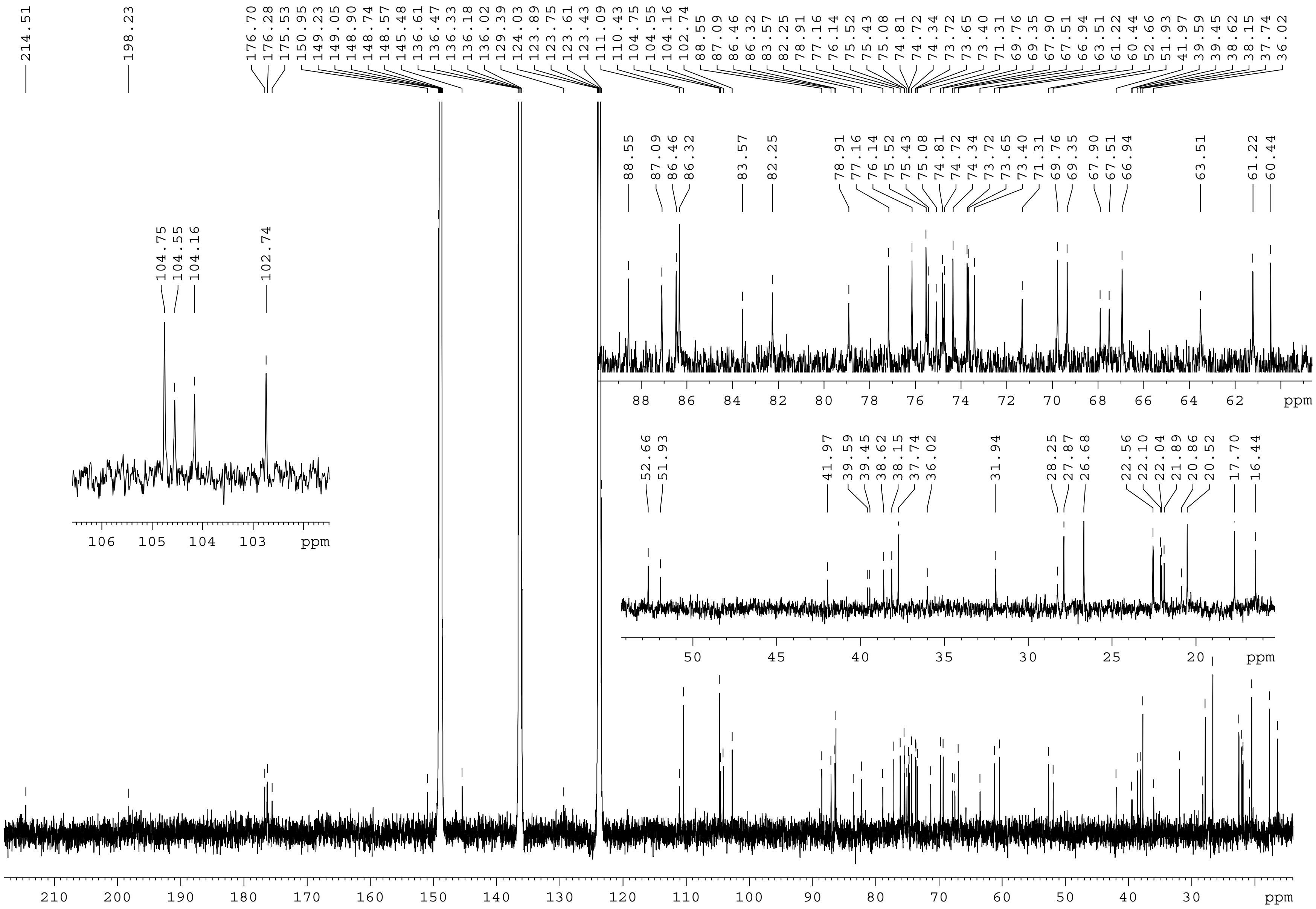

Figure S17. The ^{13}C NMR (176.04 MHz) spectrum of psolusoside P (**3**) in C_5D_5N/D_2O (4/1)

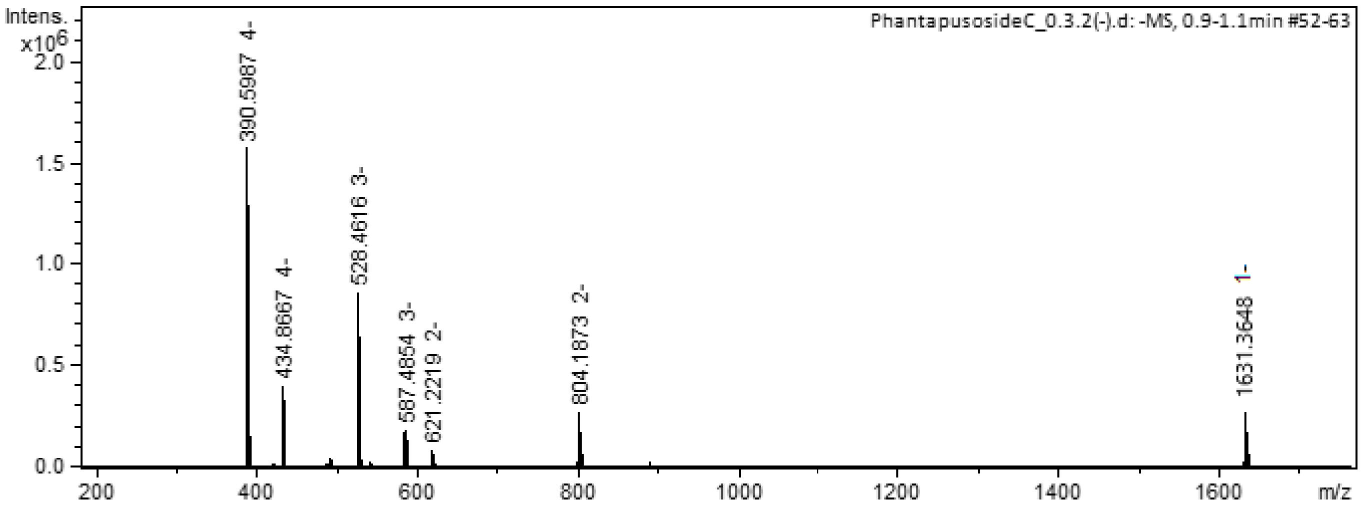

Figure S18. (–)HR-ESI-MS spectrum of psolusoside P (**3**)

Table S1. One- and two-dimensional NMR data of aglycone moiety of phantapusoside B (**2**)

Position	δ_C mult.[a]	δ_H mult. (*J* in Hz)[b]	HMBC	ROESY
1	35.7 CH_2	1.40 m		
		1.35 m		
2	26.8 CH_2	2.03 m		
		1.84 m		H-19, H-30
3	89.1 CH	3.19 dd (3.5; 11.9)	C: 1 Xyl1	H-5, H-31, H-1 Xyl1
4	39.3 C			
5	48.2 CH	0.93 d (4.3; 11.8)	C: 19	H-3, H-31
6	23.1 CH_2	1.93 m		
7	121.7 CH	5.66 m		H-15
8	143.9 C			
9	47.1 CH	3.54 brd (12.6)		
10	35.5 C			
11	22.3 CH_2	1.83 m		
		1.55 m		H-32
12	29.5 CH_2	2.23 brd (11.0)		H-21
13	56.7 C			
14	45.6 C			
15	51.9 CH_2	2.65 d (16.0)	C: 13, 16, 32	H-7, H-32
		2.32 d (16.0)	C: 14	
16	214.3 C			
17	63.4 CH	2.89 s	C: 12, 13, 16, 18, 20, 21	H-12, H-21, H-32
18	179.3 C			
19	23.9 CH_3	1.13 s	C: 1, 5, 9, 10	H-1, H-2, H-9, H-30
20	83.8 C			
21	26.1 CH_3	1.47 s	C: 17, 20, 22	H-12, H-17, H-22
22	38.2 CH_2	1.72 m		
		1.56 m		
23	22.1 CH_2	1.56 m		
		1.45 m		
24	37.8 CH_2	1.93 m	C: 23, 25, 26	
		1.88 m		
25	145.5 C			
26	110.5 CH_2	4.70 brs	C: 24, 27	H-27
		4.68 brs		H-27
27	22.1 CH_3	1.62 s	C: 24, 25, 26	
30	17.2 CH_3	1.02 s	C: 3, 4, 5, 31	H-2, H-6, H-31
31	28.6 CH_3	1.17 s	C: 3, 4, 5, 30	H-5, H-6
32	31.9 CH_3	1.17 s	C: 8, 13, 14, 15	H-15, H-17

[a] Recorded at 176.04 MHz in C_5D_5N/D_2O (4/1). [b] Recorded at 700.13 MHz in C_5D_5N/D_2O (4/1).
